# Bioelectronic Neural Interfaces: Improving Neuromodulation Through Organic Conductive Coatings

**DOI:** 10.1002/advs.202306275

**Published:** 2023-12-19

**Authors:** Wenlu Duan, Ulises Aregueta Robles, Laura Poole‐Warren, Dorna Esrafilzadeh

**Affiliations:** ^1^ The Graduate School of Biomedical Engineering UNSW Sydney NSW 2052 Australia; ^2^ Tyree Foundation Institute of Health Engineering UNSW Sydney NSW 2052 Australia

**Keywords:** bioelectronics, coatings, electrodes, neuromodulation, organic conductors

## Abstract

Integration of bioelectronic devices in clinical practice is expanding rapidly, focusing on conditions ranging from sensory to neurological and mental health disorders. While platinum (Pt) electrodes in neuromodulation devices such as cochlear implants and deep brain stimulators have shown promising results, challenges still affect their long‐term performance. Key among these are electrode and device longevity in vivo, and formation of encapsulating fibrous tissue. To overcome these challenges, organic conductors with unique chemical and physical properties are being explored. They hold great promise as coatings for neural interfaces, offering more rapid regulatory pathways and clinical implementation than standalone bioelectronics. This study provides a comprehensive review of the potential benefits of organic coatings in neuromodulation electrodes and the challenges that limit their effective integration into existing devices. It discusses issues related to metallic electrode use and introduces physical, electrical, and biological properties of organic coatings applied in neuromodulation. Furthermore, previously reported challenges related to organic coating stability, durability, manufacturing, and biocompatibility are thoroughly reviewed and proposed coating adhesion mechanisms are summarized. Understanding organic coating properties, modifications, and current challenges of organic coatings in clinical and industrial settings is expected to provide valuable insights for their future development and integration into organic bioelectronics.

## Graphical abstract

Platinum (Pt) electrode‐based neuromodulation devices hold promise for neurological disorders but face challenges of in vivo longevity and fibrous tissue formation. Organic conductive coatings are being explored for neural interfaces. This work reviews the benefits and challenges associated with organic coatings, focusing on problems with metallic electrodes and summarizing proposed coating adhesion mechanisms, providing insights for future advancement of organic bioelectronics.

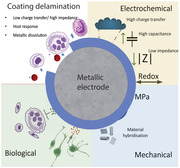

## 1 Introduction

Bioelectronic devices for modulating neural function are being increasingly integrated into clinical practice for treatment of a variety of conditions. These range from sensory disorders like hearing loss to neurological movement disorders, such as Parkinson's disease. Neuromodulating devices to treat such disorders include the cochlear implant and deep brain stimulators which largely rely on metal electrodes such as platinum (Pt).

Pt has a long history of successful use in electrodes for stimulation and recording and is stable and safe when operated below charge densities that are known to cause tissue damage.^[^ ^1^ ^]^ However, despite a long history of safe use, over time metal electrodes remain prone to deterioration and become encapsulated by non‐conductive fibrous tissue, both of which can impact on device performance.^[^ ^2^, ^3^, ^4^, ^5^ ^]^ The challenges relating to use of metal stimulating electrodes become more critical with the push for higher resolution devices requiring higher numbers of smaller electrodes to better target smaller populations of neurons. This expanding need for smaller electrodes, alongside the growing recognition that closer apposition of electrodes and neural tissue can improve longer term performance, continues to drive the search for alternative electrode materials.

Organic bioelectronics may overcome some of these challenges as their physical and chemical properties differ significantly to metals and they are more conducive to the integration of bioactive agents like anti‐inflammatory drugs and growth factors that can further improve electrode performance. There are several reviews that focus on these organic conductors and while they hold much promise for standalone bioelectronics, there remain challenges with safety and efficacy for implantable devices.^[^ ^6^, ^7^, ^8^, ^9^, ^10^ ^]^ However, organic conductors hold significant promise as electrode coatings for improving neural interfaces in the near term, with lower clinical and regulatory hurdles than standalone organic bioelectronics.

The focus of this review will be on the potential benefits of organic electrode coatings and the challenges that must be overcome to implement them in current implantable devices. Specifically, the first part of the review will outline the current issues relating to the use of metallic electrodes and will introduce organic coatings and evaluate how they could provide significant value‐add to the physical, electrical, and biological performance of such electrodes. The second section will address challenges with coatings, in particular those relating to coating stability and adhesion over long term implantation, and approaches for evaluating coating adhesion and longevity. Finally, manufacturing processes and biocompatibility considerations will be briefly addressed.

## 2 Neurostimulating Devices and Current Electrode‐Related Challenges

Neurological disorders and nerve tissue‐related diseases affect up to a billion people worldwide. Neuroprosthetic interventions aim to mitigate symptoms and restore lost sensory or neuronal functions through electrical stimulation of nerve tissue. The focus here is on implantable devices given the more stringent requirements for electrodes and the need for any coating strategy to perform under the most demanding electrical and biological conditions.

**Figure 1:**
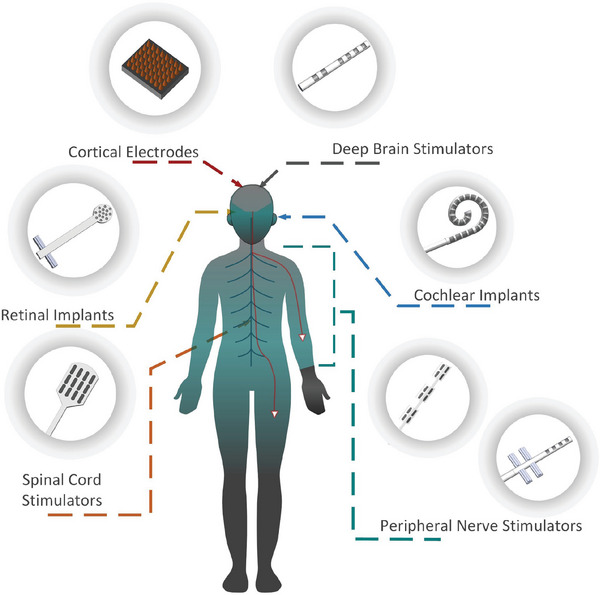
Implantable neuromodulating devices. Reproduced with permission.^[^ ^26^ ^]^ Copyright 2017, Wiley‐VCH.

Several implantable neuromodulating devices are currently used clinically including cochlear implants, auditory brain‐stem prostheses, deep brain stimulators (DBS), cortical microelectrodes, and peripheral nerve stimulating (PNS) devices, as shown in **Figure** **1**. Among these technologies, cochlear implants that return hearing sensation to profoundly deaf people are one of the most clinically and commercially successful devices.^[^ ^11^, ^12^ ^]^ DBS and PNS have also been used extensively in clinics for treating neurological and psychiatric disorders.^[^ ^13^, ^14^ ^]^ There are also many neuromodulating devices under development with future potential for clinical and commercial success including retinal implants, spinal cord stimulators and brain‐machine interfaces such as the Stentrode.^[^ ^15^, ^16^, ^17^ ^]^ These devices operate by delivering electric charge to target tissue via metallic electrodes which must sustain their performance in vivo over chronic implantation periods.

Transferring charge from metallic materials into biological tissue can be achieved via capacitive or faradaic mechanisms that are dependent on the electrode metallic material.^[^ ^18^, ^19^ ^]^ Current devices commonly use noble metals such as Pt,^[^ ^20^ ^]^ Pt‐alloys,^[^ ^21^ ^]^ and noble metal oxide based on iridium (Ir)^[^ ^22^ ^]^ as the material of choice due to their corrosion resistance, excellent conductivity, chemical stability, and biocompatibility.^[^ ^23^, ^24^, ^25^ ^]^ However, inherent material limitations relating to electrochemical, mechanical, physical, and biological performance challenge the long‐term, safe operation of neural prosthetic devices.

Key electrical limitations include low charge transfer due to inherent high impedance.^[^ ^2^, ^27^ ^]^ The electrode impedance relates to the level of charge that can be safely injected onto target tissue. Previous reviews have described that capacitive transfer mechanisms are ideal in stimulating devices, in contrast to faradic charge transfer mechanisms, which involve undesirable, irreversible redox reactions.^[^ ^18^, ^19^ ^]^ In practice, metallic electrodes like Pt transfer charge via pseudocapacitive mechanisms, where reversible faradaic reactions occur at the electrode surface while transferring charge via capacitive mechanisms. This charge transfer mechanism has been regarded as safe via delivering controlled charge‐balanced current pulses^[^ ^28^ ^]^ and within an electrochemical charge injection limit (CIL) that, once surpassed, irreversible faradaic reactions occur. The CIL for Pt has been determined experimentally for a small subset of stimulation parameters, ranging from 20 to 150 µC cm^−2^, with variations depending on the pulse leading polarity.^[^ ^29^, ^30^, ^31^ ^]^ Such range overlaps with charge densities required for effective nerve tissue stimulation, for instance ≈10 to 60 µC cm^−2^ in cochlear implants^[^ ^32^, ^33^ ^]^ and 48 to 357 µC cm^−2^ in retinal stimulation.^[^ ^34^, ^35^ ^]^ Noble metal oxides, primarily based on iridium oxide (IrOx), transfer charge through reversible faradaic mechanism involving H^+^ or OH^−^ transfer reaction and valence transition of Ir^3+^/Ir^4+^ reduction‐oxidation couple within IrOx layer.^[^ ^36^, ^37^, ^38^ ^]^ These oxide films can be formed through various methods, with the most common being sputtered deposition of iridium oxide film (SIROF), and electrochemical activation of iridium oxide film (AIROF).^[^ ^39^ ^]^ For AIROF, the charge storage capacity (CSC) and CILcan be increased substantially by cycling the electrode potential within the water window (−0.6 V – 0.8 V vs. Ag|AgCl) to promote the growth of oxide layer during each cycle.^[^ ^37^ ^]^ The CIL attained at short‐duration current pulsing ranges from 0.5–8 mC cm^−2^ depending on the electrolyte composition, pulse width, and presence of anodic bias in the waveforms during pulsing.^[^ ^38^, ^40^, ^41^, ^42^, ^43^ ^]^ It is crucial to characterize the electrical properties under conditions that match the in vivo conductivity. Comparatively, SIROF coated on Utah electrode arrays exhibits a higher electrode damage threshold than the neural damage threshold measured in physiological fluid, while the AIROF damage threshold is in close proximity to neuronal threshold.^[^ ^44^ ^]^

A key issue derived from operating around the CIL involves stimulation induced tissue damage due to pH changes as well as electrode deterioration via corrosion of the metallic substrate. While being regarded as corrosion resistant as compared to other metals, Pt electrodes can still undergo corrosion, a phenomenon more evident with high charge densities beyond safety stimulation levels.^[^ ^5^ ^]^ In addition, Pt dissolution has been detected at stimulation levels at the low range of the CIL^[^ ^30^, ^32^, ^33^, ^45^, ^46^ ^]^ even in the absence of electrical stimulation.^[^ ^5^ ^]^ IrOx electrodes demonstrated superior stability compared to pure Pt and Pt‐Ir alloy in a benchtop dissolution test.^[^ ^36^ ^]^ Nonetheless, subjecting the electrode to potential pulsing outside the water window led to electrode damage and deposition of thin IrOx layer in adjacent tissue.^[^ ^47^, ^48^ ^]^ It is still unclear to what extent this represents an issue for the electrochemical performance of metallic electrodes and the tissue response, but Pt corrosion products have been identified to perpetuate the inflammatory response in long‐term stimulation studies.^[^ ^5^, ^49^ ^]^

Biological challenges that further limit effective device operation involve poor nerve tissue integration and the host response against the implant. The biocompatibility of metallic substrates like Pt typically relates to its negligible toxicity and low reactivity with the biological environment as compared to other metals. However, Pt and other metallic electrode materials do not enable integration with healthy tissue and are ultimately subject to the host response against the implant. The end‐stage inflammatory response characterized by the formation of fibrotic tissue^[^ ^50^, ^51^, ^52^ ^]^ can challenge device operation by creating an electrical barrier between the electrode and target tissue and increasing the electrode impedance, further limiting the consistent, high‐quality neural signal recording and safe charge transfer of the device.

In terms of mechanical limitations, Pt displays notably higher stiffness, in the range of GPa, as compared to nerve tissue, with a mechanical modulus of less than 100 kPa. This mechanical mismatch can worsen the inflammatory response at the implantation site, especially during tissue movements and device micromotion and ultimately lead to loss of electrical contact and reduced therapeutic efficacy.^[^ ^24^, ^53^ ^]^ Reducing material stiffness has been shown to decrease the degree of the foreign body response.^[^ ^54^ ^]^ Thus, minimizing this mechanical mismatch via the development of softer, more pliable electrode technologies is of essence for reducing the host response against the implant and prolonging the operational lifetime of the neural prosthetic device.^[^ ^55^, ^56^ ^]^

Overall, material limitations, foreign body responses and tissue damage can maintain the device‐tissue interface under constant inflammatory conditions. Moreover, all these factors can make it difficult to decouple the effect of each factor on tissue function and device operation.^[^ ^52^ ^]^ To address these limitations, key design criteria for next‐generation devices involves miniaturization of electrodes, enhanced electrochemical properties, bioactive substrates, and more flexibles and mechanically compliant electrode materials. Ideally, devices could encourage closer apposition of electrodes and neural tissue to improve the specificity of stimulation outcomes. Traditional noble metal electrodes do not meet these criteria, but applying appropriate surface modification to the electrode surface can tailor or impart desired functionalities onto existing electrode technologies. While the field is undergoing a paradigm shift since research efforts are considering novel device designs,^[^ ^7^, ^57^, ^58^, ^59^, ^60^ ^]^ the development of a technology that can be readily applied onto existing established clinical devices is ideal as it fast‐tracks validation and translation to the clinic.

## 3 Organic Coatings for Bioelectronics: Benefits

**Figure 2:**
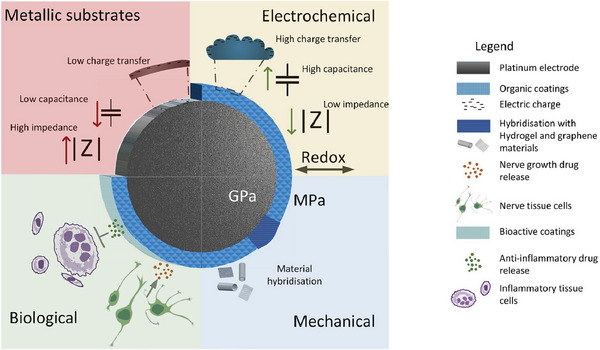
Schematic of key benefits associated with organic coatings for metal electrodes used in neuroprosthetic applications.

As discussed, one of the key challenges faced by current neuromodulation devices is sustained and stable communication between the electrode and the neural tissue. Coating metallic electrodes has the potential to improve their electrical performance as well as provide substantial benefits to the physical, mechanical, and biological interactions of devices at the tissue interface. The focus here is on organic conductors and their capacity to address the significant challenges that exist in enhancing communication at the electrode‐neural tissue interface. **Figure** **2** summarizes the benefits of organic coatings to the metallic electrode used in neuroprosthetic applications.

### 3.1 Types of Organic Coatings

The major classes of conductive organic coatings include conductive polymers, conductive hydrogels and composites that integrate conductive components with conductive or non‐conductive organic carrier materials.^[^ ^61^ ^]^ Carbon‐based materials like carbon nanotubes (CNTs) and graphene‐based materials are considered inorganic for the purposes of this review. However, as they are conductive, they have been used in composites with both conducting and non‐conducting polymers for producing organic electrode coatings.^[^ ^62^, ^63^, ^64^, ^65^ ^]^

### 3.2 Conductive Polymers

Conductive polymers (CPs) have emerged as promising coating solutions to improve the neural interface by significantly increasing CSC and CIL, while concurrently reducing the interfacial impedance. CPs also have the potential to bridge the mechanical mismatch. Organic coatings with intermediate moduli can be applied to the metal electrodes to serve as a buffer layer between rigid metal constructs and soft tissues to redistribute induced stress and moderate the mismatch.^[^ ^66^, ^67^, ^68^, ^69^ ^]^ Organic materials such as CPs and indeed other polymers exhibit lower moduli that more closely match that of soft tissue and their mechanical properties can be tailored to reduce tissue damage and enhance the overall longevity of the electrodes. They can also potentially mitigate electrode corrosion and promote favorable biological interactions between the electrode and neural tissue.

CPs have molecular backbone of alternating single bonds (σ bonds) and conjugated double bonds (π bonds) between carbon atoms that facilitate the delocalization of free carriers throughout the structure.^[^ ^70^, ^71^ ^]^ The chemical structure of the conjugated polymers allows for the incorporation of dopant molecules into the polymer backbone to facilitate the movement of charge, imparting electrical and ionic conductivity.^[^ ^72^ ^]^ The nature of dopant molecules and the polymer backbone structure, therefore, play major role in determining the overall performance of CP.^[^ ^73^ ^]^

#### 3.2.1 Dopant Molecules

Several dopants have been investigated for biomedical applications, including perchloride ions (ClO4^−^),^[^ ^74^, ^75^ ^]^ sodium p‐toluenesulfonate (pTS),^[^ ^76^, ^77^, ^78^ ^]^ tetrabutylammonium perchlorate (TBAP),^[^ ^79^, ^80^ ^]^ heparin,^[^ ^81^, ^82^ ^]^ and poly (sodium 4‐styrenesulfonate) (PSS).^[^ ^83^, ^84^, ^85^, ^86^ ^]^ The choice of dopant molecular significantly impacts the structural, chemical, electrical, and biological properties, as well as the stability of CPs. There is a general observation that as dopant size decreases, nodularity of the CP films increases. Smaller dopants like ClO_4_^−^ and pTS generally produce rougher films, whereas larger dopants such as heparin and PSS produce films with a relatively smoother surface.^[^ ^73^, ^87^, ^88^ ^]^ Furthermore, it has been observed that polymer stiffness increases with the size of the dopant ions. For instance, CP doped with PSS exhibits a modulus of 3.2 GPa, which is more than double that of ClO_4_^−^ doped CP.^[^ ^73^ ^]^ The high stiffness of PSS doped CP likely arises from the structural rigidity of PSS molecules, contributing to the formation of a relatively stiff polymer.

Variations in the nodularity of CP coatings have a significant implication on their electrical properties and biological performance. Studies indicate that smaller dopant anions lead to CP films with higher CSC and lower electrical impedance.^[^ ^73^, ^87^ ^]^ This is attributed to the greater surface roughness and nodularity in CPs doped with small anions, providing more surface area for charge transfer compared to CPs doped with large anions that have smooth surfaces. While highly nodular films are desirable for achieving optimal electrical properties, they may not be ideal for cellular interactions. The biocompatibility of CPs is influenced by the choice of dopants. When CPs degrade, the charge interactions between anodic dopants and positively charged polymer backbone are disrupted.^[^ ^73^ ^]^ Consequently, mobile dopants become free to move and diffuse into electrolyte. Thus, the toxicity of CPs is greatly influenced by intrinsic toxicity and mobility of these ions.^[^ ^87^ ^]^ In vitro cell growth inhibition assay indicates a decreasing order of toxicity: PSS > ClO_4_^−^ > pTS.^[^ ^73^ ^]^ Moreover, the surface topography of CP films is expected to affect cell response, particularly in thick films where surface island nodularity is enhanced.^[^ ^87^ ^]^ In vitro cell culture study reveals that thin film of CP doped with pTS outperform films made of other dopants,^[^ ^87^ ^]^ underscoring the significance of both cytotoxicity and surface morphology in determining the biocompatibility of CP films.

The choice of dopant molecules also greatly impacts the stability of CPs. The reversible faradaic charging mechanism, resulting from switching of CP between doped and undoped states, involves ions and electrons transport within the film.^[^ ^89^, ^90^ ^]^ As a result, the transport process of anodic dopants determines the reversibility of redox reactions, which is crucial for the application of CP in neural stimulation devices. Previous research suggests that polyanions like PSS become entrapped within the CP matrix due to their large molecular weight and entanglement with polymer chains. As such, ion transport in PSS doped CPs is primarily based on Na^+^ cation transport, whereas small anodic dopants such as pTS and ClO_4_^−^ can move freely during switching.^[^ ^73^, ^87^, ^90^ ^]^ This transport phenomenon has important implications on CP film stability. Smaller dopants, while enhancing the electrical and biological properties of CPs, are prone to displacement by ions in the electrolyte during chronic current pulsing, especially in an in vivo setting with active body fluid circulation capable of washing away those diffused dopants. In contrast, CPs doped with larger anions, immobilized within the CP structure, generally exhibit greater stability and are suitable for coatings on chronic neurostimulation electrodes.^[^ ^89^, ^91^ ^]^

**Figure 3:**
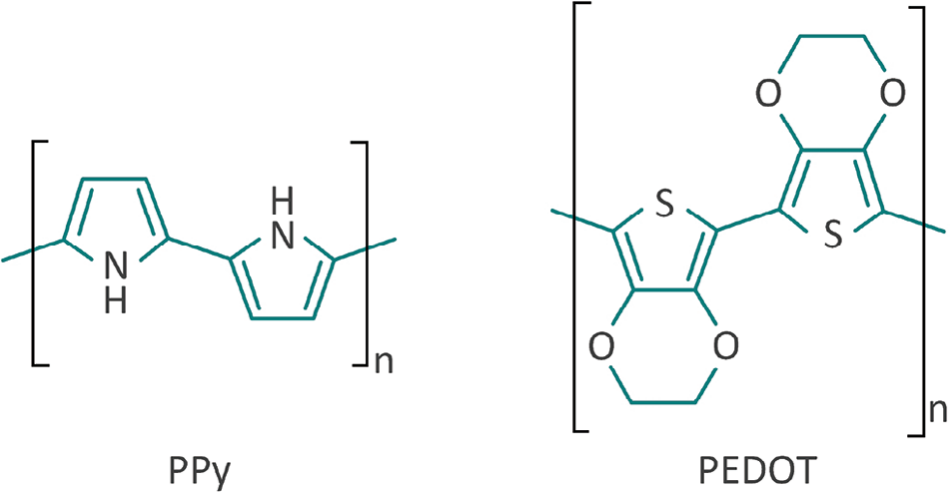
Chemical structure of polypyrrole (PPy) and poly (3,4‐ethylendioxythiophene) (PEDOT).

Various CPs have been explored for coating neural electrodes, with polypyrrole (PPy) and poly (3,4‐ethylendioxythiophene) (PEDOT) being the most widely investigated.^[^ ^75^, ^92^ ^]^ The chemical structure of these CPs is illustrated in **Figure** **3**.

#### 3.2.2 Polypyrrole

PPy has been studied in neural interfacing electrodes and literature reports support its biocompatibility,^[^ ^78^, ^93^, ^94^, ^95^ ^]^ stimulus‐responsive properties,^[^ ^82^, ^96^ ^]^ chemical stability^[^ ^97^ ^]^ and high conductivity in physiological environments.^[^ ^62^, ^75^, ^98^, ^99^, ^100^ ^]^ The application of PPy on neural electrodes for promoting neural cell growth, establishing connection with native tissue, and controlled drug delivery for use have also been investigated. PPy:pTS containing neurotropin‐3 was applied to cochlear implant electrode and implanted in Guinea pigs. After two weeks of stimulation, the animals with electrically stimulated neurotrophins loaded electrodes had lower electrically‐evoke auditory nerve response threshold and greater spiral ganglion neurons density compared to non‐implanted cochlea and polymers without neurotrophins.^[^ ^78^ ^]^ Another study conducted by Evans et al., incorporated brain‐derived neurotrophic factor (BDNF) into PPy:pTS to investigate the passive and dynamic release of BDNF through diffusion and electrical stimulation. The stimulated polymers released 1.75‐fold more BDNF compared to unstimulated polymers, and the presence of BDNF significantly increased the neurite outgrowth from spiral ganglion neuron explants.^[^ ^93^ ^]^

The capability of PPy for incorporating bioactive molecules and drugs has shown great promise toward developing biocompatible intracortical electrodes. Chronic neural recording electrodes fails after implantation primarily due to neuro‐inflammatory response that leads to glial sheath formation and neuronal loss at the site of implant.^[^ ^101^ ^]^ PPy doped with dexamethasone, an anti‐inflammatory drug, has shown to reduce inflammation by inhibition of astrocyte proliferation compared to control in an in‐vitro study.^[^ ^102^ ^]^ A different approach involved using negatively charged biotin as dopant for PPy formation to achieve controlled drug delivery. Biotin‐doped PPy was electrodeposited onto gold and incubated with streptavidin, allowing attachment of biotinylated drugs such as nerve growth factor. Drug release was triggered by electrical stimulation that resulted in reduction of PPy backbone.^[^ ^94^ ^]^ Additionally, Cui et al., focused on surface modification of neural probes with peptide incorporated into CP to improve the long‐term functionality of the devices. Laminin fragment CDPGYIGSR and RNIAEIIKDI have been studied as dopants for PPy to modify gold substrate. The modified surface consistently shows lower impedance, larger charge transfer capacity, longer primary neurite outgrowth, and lesser astrocyte adhesion compared to unmodified surface.^[^ ^103^ ^]^ Gold electrodes were coated with PPy/DCDPGYIGSR and incubated in distilled water for up to 7 weeks. The soft, fuzzy morphology and the reversible redox nature of the coatings effectively increased the surface area of the electrode, leading to reduced impedance and phase at the biologically relevant frequency of 1 kHz, increased CSC and provided high density site for biological interactions with neural cells.^[^ ^99^ ^]^ Further optimization of neural interface involves formation of porous PPy loaded with nerve growth factor. Such topography allows for enhanced entrapment and release of bioactive molecules, ultimately leading to enhanced cellular viability and neurite extension.^[^ ^104^ ^]^ However, the chemical structure of the CP film altered, and the impedance increased after only one week of immersion indicating more stable CP matrices are needed to ensure long‐term functionality.^[^ ^95^ ^]^

Despite numerous reports of PPy as a coating material for neural interfacing electrodes, issues associated with its stability may limit its use in biomedical applications. During the polymerization of pyrrole, the chemical exhibits three possible polymer chain formation through either σ‐ σ, σ‐β, and β‐β coupling.^[^ ^105^ ^]^ The presence of σ‐β and β‐β coupling along the polymer chain leads to structural disorder, limiting the electroconductivity of the polymer, and has been implicated as the primary site of polymer breakdown due to over‐oxidation of the polymer.^[^ ^85^, ^86^ ^]^ It has been reported that PPy:PSS loses 95% of its original electroactivity after 16 h of constant potential polarization in phosphate buffer solution^[^ ^106^ ^]^ and 50% of its original CSC after 50 cycles of potential cycling,^[^ ^86^ ^]^ attributed to the instability of the PPy film.

PEDOT, as illustrated in Figure 2, has a dioxyethylene bridging group across the 3‐ and 4‐ positions of the heterocycles which blocks the possibility of σ‐β’ coupling and consequently it has higher electrochemical stability in comparison with Ppy.

#### 3.2.3 Poly (3,4‐ethylendioxythiophene)

Polythiophene, particularly its derivative poly (3,4‐ethylendioxythiophene) (PEDOT) has been widely investigated for applications in neural interfacing electrodes. Being relatively stable compared with PPy, PEDOT has become the dominant choice for coating neural electrodes in the past decade. It possesses several desirable properties including significantly reduced interfacial impedance, higher CSC, higher CIL, improved charge transfer mechanism, and evidence of biocompatibility.

PEDOT doped with LiClO4, sodium benzenesulfonate (BS), pTS, sodium dodecylbenzene sulfonate (DBS), and PSS have been investigated for neural interfacing applications.^[^ ^73^ ^]^ Compared with bare Pt electrodes, PEDOT coatings significantly reduced the interfacial impedance in saline solution over frequency ranges between 0.1 Hz to 1 kHz. The impedance varied among the different dopants, with LiClO4 showing the lowest impedance and increasing for pTS, BS, DBS, and PSS doped coatings. Phase plots indicated a capacitive charge transfer characteristic of CP at frequency below 10 Hz, becoming purely resistive as frequency approached 1 kHz, whereas Pt electrodes are predominantly capacitive at all frequencies toward 10 kHz.

PEDOT:PSS was electrochemically deposited on thin film Pt stimulation electrodes to evaluate its performance for chronic stimulation.^[^ ^107^ ^]^ Significant improvement in the electrochemical stability was observed, with 89% of its original electroactivity conserved after constant potential polarization and repetitive potential cycling under the same conditions as used for PPy:PSS.^[^ ^86^, ^106^ ^]^ PEDOT:PSS coatings also decreased impedance modulus of Pt electrodes by 2–3 orders of magnitude at frequency range of 1–100 kHz, and this decrease was more pronounced at lower frequency ranges between 1–200 Hz. The low impedance magnitude was attributed to increased surface area and high ionic conductivity of PEDOT film.

PEDOT has also been shown to effectively lower impedance and improve the recording quality of neural recording electrodes. In a 7‐day implantation study, PEDOT‐coated gold electrodes exhibited a tenfold reduction in impedance compared to unmodified electrodes at 1 kHz.^[^ ^108^ ^]^ Over a longer 6‐week period, PEDOT‐coated sites demonstrated better signal‐to‐noise ratio and viable unit potential counts compared to control sites.^[^ ^109^ ^]^ However, both PEDOT and uncoated sites experienced tissue response, resulting in an average impedance increase of 700 kΩ at 1 kHz, which diminished the initial benefits of lower impedance from the PEDOT film.

The application of PEDOT coating significantly enhances the CSC and CIL^[^ ^110^ ^]^ of neurostimulation electrodes. In comparison to bare Pt microelectrodes, PEDOT:PSS coated Pt electrodes demonstrated approximately three times higher CIL and up to 9.5 times higher CSC.^[^ ^111^ ^]^ PEDOT also surpasses IrOx electrodes in terms of CSC, exhibiting more than double its value, while its CIL of 2.3 mC/cm^−2^ is comparable to that of IrOx.^[^ ^112^ ^]^ PEDOT doped with Tetraethylammonium tetrafluoroborate (TEABF4) further enhanced the CSC of deep‐brain‐stimulation electrodes by 3.8 times when processed in propylene carbonate.^[^ ^113^ ^]^ For retinal implants, PEDOT:pTS coated Pt microelectrode arrays displayed higher CIL, ranging from 1.2–3.9 mC cm^−2^ in saline and 1.5–2.6 mC cm^−2^ in protein‐loaded solutions, compared to Pt electrodes which range from 0.08–0.12 mC cm^−2^ in saline and 0.05–0.07 mC cm^−2^ in protein media.^[^ ^77^ ^]^ During biphasic pulse stimulation, PEDOT also displayed a much lower initial ohmic voltage excursion and followed by a flattened polarization. This improved charge injection mechanism is especially favorable for neural stimulation as it is able to push current at much lower voltage amplitude, consequently, reduce the formation of irreversible electrochemical reaction by‐products, and lower the overall battery consumption of the devices.^[^ ^107^, ^112^ ^]^ PEDOT therefore offered higher capability to convert those charges that stored in the film into injected pulse.

Implanting neural electrodes triggers an inflammatory response against foreign material, leading to tissue reactions and decreased device performance. To address this issue, research has explored PEDOT as a means to incorporate therapeutic functions and enhance tissue‐device connections. PEDOT nanotubes with well‐defined structure were manufactured by electrospinning biodegradable poly(L‐lactide) (PLLA) or poly(lactide‐co‐glycolide) (PLGA) onto gold electrodes, followed by electrochemical deposition of PEDOT around the nanofiber structures. Dexamethasone was incorporated into nanofibers, which could be released either through passive diffusion from PLLA/PLGA degradation or actively by applying electrical potential.^[^ ^114^ ^]^ PEDOT was also investigated in combination with extracellular matrix molecules and nerve growth factors (NGFs) to enhance cell attachment and promote neurite outgrowth. Laminin peptides DEDEDYFQRYLI and DCDPGYIGSR doped with PEDOT significantly improved the cell adherence and neurite outgrowth.^[^ ^115^ ^]^ NGFs entrapped in laminin peptide doped PEDOT:pTS coatings produced neurite outgrowth comparable to control films where NGF was directly supplied via the medium.^[^ ^116^ ^]^ However, the inclusion of large biomolecules such as peptides can significantly affect the physiochemical properties of CP film. The film produced is rather softer and electrochemically unstable due to incomplete polymerization, suggesting the need for smaller biomolecular dopants to minimize these effects.

Despite the many advantages of PEDOT as an electrode coating, its mechanical properties are many orders of magnitude higher than those of neural tissues. Attempts to address this mechanical mismatch have focused on the development of softer organic coatings such as those based on hydrogels.

### 3.3 Conductive Hydrogel

The intrinsic dissimilarity between rigid neural electrodes and soft living tissues poses great challenges in developing biocompatible, effective, and chronically stable bioelectronic interfaces.^[^ ^117^ ^]^ Hydrogels, which constitute a group of hydrophilic polymer chains capable of holding a large amount of water and the capacity to tailor mechanical, electrical, and biological properties, have emerged as promising candidates to address the limitations of metal electrodes.^[^ ^118^ ^]^ Hydrogel mechanical properties provide a buffer layer between hard electrodes with moduli in the GPa range and soft tissues with moduli in the Pascal to kPa range, potentially reducing tissue response.^[^ ^66^ ^]^ Their open mesh structure also allows ion diffusion through the matrix, resulting in minimal increases in electrode impedance.^[^ ^119^ ^]^ Extensive research has advanced tailoring of hydrogel mechanical properties and imparted electroactivity to engineer conductive hydrogels (CHs) as next‐generation materials suitable for neural interfacing applications.

Incorporating CP into hydrogels enables the preservation of desirable electrical and biological properties while matching the stiffness of neural electrodes to soft tissues. The 3D hydrogel network facilitates the growth of CP during polymerization, leading to enhanced electrochemical properties of bioelectrodes due to increased surface area.^[^ ^120^ ^]^ For applications in neural stimulation electrodes, the rate of biphasic stimulation is too fast to support the slow ionic flux during redox reaction.^[^ ^121^ ^]^ The CH thus enables fast double‐layer formation across the large surface area, further improving the charge transfer mechanism. Moreover, the hydrophilic mesh provides means for incorporating water‐soluble drugs and growth factors into the hydrogel matrix, addressing the challenges associated with stability when functionalizing CP with bioactive molecules.^[^ ^69^ ^]^

Integrating two dissimilar polymer systems to achieve an interpenetrating network (IPN), where the hydrogel is homogeneously occupied by CP while maintaining electrode spatial resolution is challenging. The fabrication of CP typically involves using freely mobile dopants to balance the charge on the polymer backbone. The electropolymerizing takes place at the metallic site of the electrodes where free radicals are readily available.^[^ ^122^ ^]^ As the result, CP occupies only a small portion of the hydrogel on the underlying substrate, potentially causing displacement of hydrogel coatings.^[^ ^123^ ^]^

**Figure 4:**
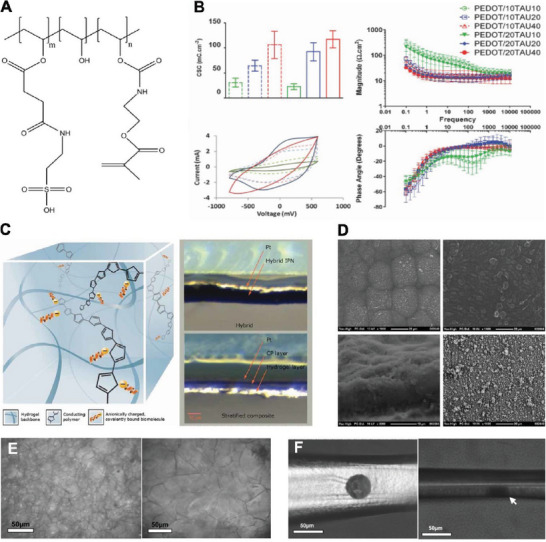
A) Chemical structure of taurine substituted PVA. Reproduced with permission.^[^ ^124^ ^]^ Copyright 2017, Wiley‐VCH. B) CSC, cyclic voltammetry (CV), and electrochemical impedance spectrometry (EIS) results of Pt electrodes coated with CH produced from PEDOT grown in PVA‐Taurine hydrogel. Increased Taurine substitution results in increased growth of PEDOT and increased electrical properties. Reproduced with permission.^[^ ^124^ ^]^ Copyright 2017, Wiley‐VCH. C) Schematic of interpenetrating CH network where CP is grown within the hydrogel structure along the crosslinked dopant molecules (left), and comparison of material formed by covalently bounded dopant and material produced from mobile dopant (right). Reproduced with permission.^[^ ^123^ ^]^ Copyright 2012, Wiley‐VCH. D) SEM of surface topography of PEDOT grown in PVA‐heparin (top left), heparin (top right), cross‐section of PEDOT in PVA‐heparin showing a throughout, integrated material growth (bottom left), compared to conventional PEDOT:pTS (bottom right). Reproduced with permission.^[^ ^123^ ^]^ Copyright 2012, Wiley‐VCH. E) Optical image of N_2_‐frozen (left) and freezer‐frozen (right) hydrogel showing a more porous microstructure (≈50 um size). Reproduced with permission.^[^ ^126^ ^]^ Copyright 2004, Wiley‐VCH. F) Optical image of PPy grown in lyophilized hydrogel on a Michigan type silicone probe (left), and lateral view of coating showing growth of PPy toward the surface without spreading out (right). Reproduced with permission,^[^ ^126^ ^]^ Copyright 2004, Wiley‐VCH.

Research in this field has focused on directing the growth of CP within hydrogel by controlling the nucleation point. One strategy optimized loading of pre‐polymerized PEDOT within the hydrogel to impart bulk conductivity and facilitate nucleation.^[^ ^122^ ^]^ Another approach is in chemical modification of base hydrogel backbone with sulfonate or taurine doping groups providing a high‐density “fixed” dopant pathway that guides the growth of CP throughout the hydrogel.^[^ ^124^ ^]^ A higher degree of taurine substitution promotes the presence of PEDOT within the CH system (**Figure** **4**A), directly improving electroactivity, leading to increased CSC and reduced impedance (Figure 4B).^[^ ^124^ ^]^ Finally, hybrid hydrogels formed by covalently modifying poly(vinyl alcohol) (PVA) with methacrylated heparin (Figure 4C), enabled formation of large and more uniform nodular aggregations of CP than conventional homogeneous PEDOT (Figure 4D).^[^ ^123^ ^]^

In another approach, a co‐polymer hydrogel consisting of dimethylacrylamide (DMAA), UV‐reactive 4‐methacryloyloxy benzophenone (MABP), and sodium 4‐styrenesulfonate (SSNa), which served as the dopant molecule, was developed to permanently integrate PEDOT into hydrogel scaffolds.^[^ ^125^ ^]^ Lyophilized hydrogel was also explored for directing the growth of PPy through the hydrogel coating without impacting adjacent electrode sites. In such microporous hydrogel, CP is more densely packed due to larger pore size compared to intact hydrogel (Figure 4E), growing straight toward the hydrogel surface and retaining the spatial resolution of the electrode (Figure 4F).^[^ ^126^ ^]^

Neural electrodes modified with CH have demonstrated a significant electrochemical advantage, exhibiting higher CSC, CIL, and lower interfacial impedance compared to unmodified metal electrodes and those modified with homogeneous CPs, both in vitro and in vivo.

CH developed by growing PEDOT:pTS within heparin‐modified PVA hydrogel coated on cochlear electrode arrays had significantly increased CSC from 13 to 124 mC cm^−2^ compared with Pt. The CIL of Pt assessed at pulse width from 0.025 to 0.8 ms after CH modification increased from 0.005 to 0.175 to 0.085–2.418 mC cm^−2^ respectively.^[^ ^127^ ^]^ The heparin chain in the hydrogel served as a dopant, enabling formation of CP through the hydrogel layer and preserving the conductive sites above each isolated electrode. In another study, CH fabricated by growing PEDOT:pTS in PVA‐Tau hydrogel was coated on paddle and ring Pt electrodes, leading to significantly lower impedance magnitude throughout 1 to 100 kHz frequency spectrum. Although demonstrating significantly higher CSC and CIL, there was higher variability.^[^ ^128^ ^]^ The same CH formulation was used on stainless‐steel electrodes for peripheral nerve blocking using high‐frequency stimulation, yielding an improved CSC by at least two orders of magnitude and reduced the impedance across all frequencies when compared to unmodified electrodes.^[^ ^76^ ^]^

**Figure 5:**
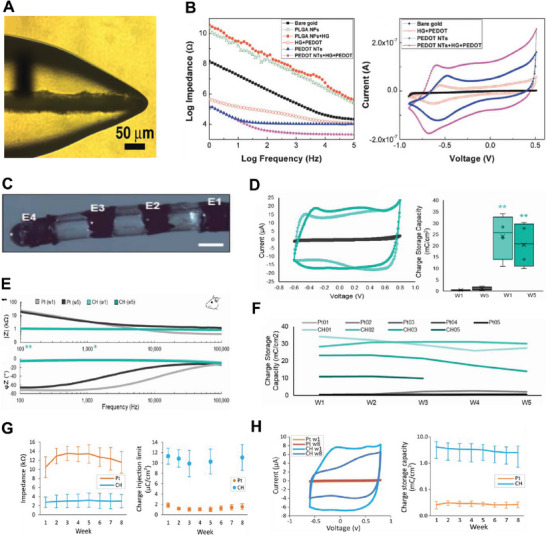
A) Optical image of PEDOT deposited on the electrode site showing the growth of PEDOT through the hydrogel scaffold toward the surface. Reproduced with permission.^[^ ^129^ ^]^ Copyright 2009, Wiley‐VCH. B) EIS and CV of bare gold electrodes, and electrodes modified with PLGA nanofibers, nanofibers + alginate hydrogel, nanostructured PEDOT, smooth PEDOT in hydrogel, and nanostructured PEDOT in hydrogel. Reproduced with permission.^[^ ^129^ ^]^ Copyright 2009, Wiley‐VCH. C) Optical image of cochlear implant array modified with CH coating. Reproduced with permission.^[^ ^121^ ^]^ Copyright 2020, IOP Publishing. D) In‐vivo CV curves showing the CSC of CH‐modified electrodes was significantly greater than that of Pt electrodes. Reproduced with permission.^[^ ^121^ ^]^ Copyright 2020, IOP Publishing. E) In vivo EIS data showing the impedance magnitude of CH‐modified electrodes was significantly lower than Pt electrodes. Reproduced with permission.^[^ ^121^ ^]^ Copyright 2020, IOP Publishing. F) CSC of CH coated electrodes stayed stable over the implantation period. Reproduced with permission.^[^ ^121^ ^]^ Copyright 2020, IOP Publishing. G) Voltage transient (VT) impedance (left) and CIL (right) of rat DBS array modified with CH measured in vivo. The VT impedance of CH modified electrodes was significantly lower than that of Pt electrodes, and CIL was significantly higher than that of Pt electrodes across all electrode contacts for the duration of the study. Reproduced with permission.^[^ ^130^ ^]^ Copyright 2021, Frontiers. H) CSC of both electrode groups derived from CV curves: the mean CSC of CH‐modified electrodes was significantly higher than that of Pt electrodes. Reproduced with permission.^[^ ^130^ ^]^ Copyright 2021, Frontiers

Nanostructured PEDOT:LiClO_4_ electropolymerized in alginate hydrogel was encapsulated on gold neural recording electrodes (**Figure** **5**A). The coating resulted in two orders of magnitude increase in CSC and a decrease in interfacial impedance at 1 kHz (Figure 5B).^[^ ^129^ ^]^

The electrochemical advantages of CH coating were also evident in vivo. CH formed by PEDOT grown in PVA‐Tau was coated on Pt electrodes (Figure 5C) and implanted in rat cochlea over five‐week stimulation periods. Both CSC and CIL of CH coated electrodes were significantly higher than those of Pt electrodes (Figure 5D) without significant change over time (Figure 5F). And the in vivo impedance magnitude of CH coated electrodes remained significantly lower than uncoated Pt electrode in the lower frequency range throughout the implantation period (Figure 5E).^[^ ^121^ ^]^

Similar CH variants tailored with lower swelling behavior through co‐polymerization with methacrylated PVA (PVA‐MA), were coated on DBS electrodes and implanted in rat brain tissue. CH‐coated electrodes demonstrated significantly lower voltage transient impedance (Figure 5G), which stays stable over the implantation period, and consistently higher CIL (Figure 5G) and CSC (Figure 5H) compared to Pt electrodes.^[^ ^130^ ^]^

CH coatings formed by combining PEDOT:PSS and arginine‐glycine‐aspartic acid (RGD)‐functionalized alginate hydrogel maintained consistently lower impedances of cochlear model implant throughout 6 months period, while bare Pt electrodes experienced impedance increase post‐implantation.^[^ ^131^ ^]^ An interpenetrating network of PEDOT:PSS and acrylic acid (AA) modified poly vinyl alcohol and poly acrylic acid (PVA/PAA) was applied on Pt‐based optical fiber electrode (optrode) arrays and implanted in rat hippocampus. The CH‐modified optrode displayed significantly lower impedance at 1 kHz in vivo and improved signal‐to‐noise ratio (SNR) compared to unmodified sites over the course of a four‐week implantation period.^[^ ^132^ ^]^

The feasibility of CH as coating materials for improving the biocompatibility of neuroprosthetic devices and incorporating biomolecules to benefit the biological environment of the device‐tissue interface has been demonstrated. A coating consisting of arginine‐glycine‐aspartic acid (RGD)‐functionalized alginate hydrogel and PEDOT was developed to act as an artificial extracellular matrix, supporting neural cell growth and delivering brain‐derived neurotrophic factor (BDNF) sustainably without affecting electrical properties.^[^ ^131^ ^]^ The concept of a “living” electrode containing viable cells has also been proposed. Such systems comprise a cell‐laden layer embedded within a degradable hydrogel coating, facilitating the formation of extracellular matrix within the electrode construct without compromising electrical properties and supporting device integration into tissue environments.^[^ ^133^ ^]^ The application of CH coatings on neural electrodes demonstrated their potential as a versatile engineering platform for minimizing inflammation, foreign body response, and promoting neuron survival post‐implantation.

CHs have also found their applications in other electroresponsive tissues, including the heart.^[^ ^134^ ^]^ Many cardiac diseases disrupt the electrical signal of the heart wall. Cardiac patches made of CHs have shown promise in restoring cardiac function and repairing damaged tissue.^[^ ^135^ ^]^ Polymerization of pyrrole doped with phytic acid on prefabricated chitosan films led to the formation of a conductive cardiac patch. This patch demonstrates high electroactivity and low surface resistivity after a two‐week incubation in a physiological medium.^[^ ^135^ ^]^ The immobilization of phytic acid in the CP prevents electrical degradation, thus prolonging stability.^[^ ^136^ ^]^ Ex vivo experiments reveal that the conductive patch significantly increases conduction velocity of the infarcted area, both in cardiac tissue slice and whole heart.^[^ ^135^ ^]^

When a liquid form of PPy‐chitosan hydrogel is injected at the site of myocardial infarction, it reduces the QRS interval and enhances the transverse activation velocity, indicating improved electrical conduction between infarcted cardiomyocytes and healthy intact tissue.^[^ ^138^ ^]^ Furthermore, injectable CHs also demonstrate potential to reduce the myocardial threshold voltage for pacemaker stimulation. Local fibrosis and glial scarring can increase tissue impedance and reduce myocardial conductivity, making pacemaker battery consumption a detrimental challenge. Injection of poly‐3‐amino‐4‐methoxybenzoic acid‐gelatin (PAMB‐G) into the electrode‐tissue interface significantly decreases impedance and increases myocardial cell membrane voltage, facilitating charge transfer and improving cardiac pacemaker efficiency.^[^ ^139^ ^]^ The versatility of CH in cardiac tissue interfacing applications, driven by its biocompatibility, electrical properties, and superior coupling properties with native tissue, holds great promise for advancing cardiac tissue engineering approach and treating heart failure and other electroresponsive tissue‐related diseases.

### 3.4 Carbon‐Based Coatings

CNTs and graphene demonstrate high electrical conductivity and thermal stability and have been applied to neural interfacing electrodes to enhance their electrochemical properties.^[^ ^139^, ^140^, ^141^, ^142^ ^]^ The intrinsically large surface area and high conductance of carbon nanomaterials significantly reduce the electrode impedance, making electrodes highly polarizable to accommodate a large amount of injected charge on the double‐layer before faradaic reactions take place.^[^ ^143^ ^]^ Carbon‐based materials tend to aggregate due to their hydrophobic nature, leading to cytotoxicity concerns and potential tissue damage. The interactions between carbonaceous materials with cells and their impact on the biocompatibility have been extensively reviewed but a definitive conclusion is hard to draw due to various physiochemical structures of the nanomaterials. Factors such as shapes, time of exposure, and concentrations of the nanomaterials are the main concerns.^[^ ^144^, ^145^, ^146^, ^147^ ^]^ While these materials are not considered as organic, they are conductive and often incorporated into organic materials to impart conductivity and form a composite coating.^[^ ^61^ ^]^

### 3.5 Composite Materials

With significant advancements in the techniques to synthesize composites, there has been a growing emphasis on the integration of different material components for producing composites. This process is carried out with the aim of utilizing the most desirable characteristics of the individual materials in the combined structure. Thus, conductive composites aim to improve combinations of electrical, physical, mechanical and biological properties.

Numerous studies have explored the effects of integrating metals and metal oxides, such as gold (Au), Pt, and Ir, in composite coatings applied to neurostimulation electrodes. However, this section focuses on analyzing composites that consist of CPs and carbon‐based materials, specifically CNTs and graphene derivatives.

Several examples of carbon‐based materials incorporated into PPy to improve the electroactivity of the film have been reported. Multi‐walled carbon nanotubes (MWCNTs) copolymerized with PPy:PSS onto Pt improved CSC which was retained after repetitive potential cycling by 50% compared to PPy:PSS film.^[^ ^62^ ^]^ Graphene oxide was also incorporated into PPy:PSS through electrostatic interaction to produce composite film on Pt electrode. The coating resulted in two orders of magnitude increase in CSC and lowered the impedance of electrode by 90% at 1 kHz compared to Pt counterpart.^[^ ^98^ ^]^ The improved electrical property of the film was attributed to rougher surface with increased effective surface area, reducing impedance and increasing conductivity.

Electrode performance of PEDOT:PSS‐graphene oxide (GO) composites electrodeposited on gold electrodes were evaluated as durable neural micro‐electrode coatings. Higher electrochemical stability was reported for PEDOT:PSS integrating reduced graphene oxide (rGO) compared to PEDOT:PSS due to higher conductivity of rGOs providing fully active charge transfer sites along with the porous and bulky composite structure. Despite the improved electrochemical performance, there may be obstacles that originate from the over‐oxidation of GO during the synthesis process. This reduces the restoration of conjugated carbon structures when it eventually transforms into rGO. The electrical conductivity of rGO is largely determined by the quality of its sp2‐conjugated carbon bonds. Therefore, it is crucial to thoroughly develop GO and rGO materials with minimal structural defects to achieve the highest possible conductivity.^[^ ^148^ ^]^

The other complexity relating to CNT is their biocompatibility. Despite their high electrochemical conductivity and mechanical properties, their cytotoxicity, particularly in the long term, remains a concern. The CNTs cytotoxicity is controversial topic, particularly regarding their size and ability to penetrate cells, inducing biological reactions. The absence of globally agreed standards for testing and CNTs homogeneity is a significant challenge when assessing the suitability of CNTs when considered as bioelectrode coating. The findings reported from cytotoxicological studies are greatly impacted by multiple factors, including particle size, medium type, agglomerate size, and surface morphology. These factors, in combination with the experimental parameters and intended applications, play a crucial role in cytotoxicity evaluation of CNTs. Therefore, it is crucial to adopt a case‐specific methodology to conduct a thorough assessment of electrodes coated with CNTs.^[^ ^149^ ^]^

Studies reported that integrating CNTs within composites is potentially beneficial, enabling exploitation of their electrical performance as well as using their drug loading capacity to integrate bioactive components in the coatings. Single‐wall carbon nanotubes (SWCNTs)^[^ ^150^ ^]^ and MWCNTs^[^ ^151^ ^]^ have been combined with PEDOT to create composite coatings that enable controlled drug release during neural stimulation. The combination of PEDOT with CNTs improved the drug loading capacity owing to the porous structure of nanotubes, as well as enabling fast drug release due to the high conductivity of carbon nanotubes through electrical stimulation. In vivo studies demonstrated that drug‐loaded PEDOT‐coated electrodes reduced axonal damage, neuronal cell death, and inflammation compared to non‐coated electrodes.^[^ ^151^ ^]^

### 3.6 Organic Electrode Coating for Corrosion Protection

A key advantage of organic coatings that has had limited attention is the potential for enhanced protection against corrosion. The main body of work in this area encompasses non‐biomedical applications.

CP may prevent metal corrosion and dissolution, inducing or maintaining the formation of protective film layers^[^ ^152^ ^]^ via several mechanisms. First, a strongly adherent CP coating exhibits a barrier effect on coated metal to limit the ingression of oxygen, water, and other ions from approaching electrodes.^[^ ^153^, ^154^ ^]^ Second, CP may induce or maintain the formation of passive oxide layer on metal surface, thus reducing the rate of metal corrosion.^[^ ^155^, ^156^ ^]^ Third, CP with its intrinsic redox property may mediate the electrons generated by metal dissolution and consumed by oxygen reduction, as shown in the equations below:

(1) $$M\to M^{2+}+2e^{-}$$

(2) $$CP_{ox}+2e^{-}↔CP_{red}$$

(3) $$\frac{1}{2}O_{2}+H_{2}O+2e^{-}\to 2OH^{-}$$

The CP may therefore act as a redox catalyst on the metal surface that will be reduced as a consequence of metal surface passivation and reoxidized by oxygen reduction. As a result, CP may promote the cathodic oxygen reduction reaction, which is one of key reactions taking place during the cathodic phase of biphasic stimulation,^[^ ^157^, ^158^, ^159^ ^]^ on the polymer surface, rather than the metal‐polymer interface. This local separation of metal oxidation reactions and oxygen reduction reactions has the potential to maintain the oxide film formed on the metal surface, limiting the reduction product OH^−^ crossing metal‐CP interface, preventing the cathodic dislodgement of metal lattice, thereby limiting metal dissolution.^[^ ^97^, ^160^, ^161^ ^]^

PPy has been utilized for corrosion protection in various non‐biomedical metal surface applications. In the electropolymerization process of PPy on copper (Cu) and copper‐nickel (Cu‐Ni) substrates, the presence of sulfur,^[^ ^162^ ^]^ oxalate,^[^ ^163^ ^]^ or aqueous phosphate‐containing solutions^[^ ^164^ ^]^ facilitated the initial formation of a passivation layer composed of copper oxide and hydroxide.^[^ ^165^ ^]^ This passive layer significantly reduced Cu dissolution while maintaining conductivity, enabling the formation of a well‐adhered PPy film. Corrosion tests conducted in sodium chloride solution revealed a higher initial open‐circuit potential (OCP) for PPy‐coated Cu compared to pure Cu. The anodic polarization curve exhibited lower current and higher corrosion potential relative to uncoated Cu. After 20 h of incubation, the OCP of the coated Cu decreased and approached that of Cu after 40 h, possibly due to water intake and transport of chloride ions across the polymer film. Observations from SEM images after 7 days of incubation indicated the absence of localized accumulation of corrosion products on the PPy‐coated Cu, suggesting uniform corrosion that was not initiated at defects within the PPy film.

MWCNTs were added to PPy to create composite films for corrosion protection on copper‐zinc brass alloy surfaces.^[^ ^166^ ^]^ The incorporation of nanotubes resulted in increased resistance to corrosion by hindering the movement of corrosive ions. The level of resistance was dependent on the loading of nanotubes, with polymers containing more than 1% nanotube loading showing inadequate distribution within the film.

Other CP that have been investigated metal corrosion protection include polyaniline (PANi)^[^ ^154^, ^168^, ^169^, ^170^, ^171^ ^]^ and PEDOT.^[^ ^160^, ^161^ ^]^ PANi provides protection by releasing anions to form a passivating salt, creating a second protective layer due to its redox properties. When the coating is damaged, local corrosion triggers metal oxide formation at those sites. The surrounding CP^[^ ^171^ ^]^ is reduced through a redox reaction with PANi upon damage, and it is later re‐oxidized by oxygen in the environment, restoring its oxidizing power.^[^ ^167^, ^168^, ^169^ ^]^ PANi‐coated films on steel, iron, copper, and other substrates exhibit stable high OCP over extended periods, demonstrating their corrosion protection capability. Visual observation revealed that samples with corrosion suppression develop an oxide layer over the bare metal, supporting the proposed mechanism that anodic protection improves corrosion protection by inducing a shift in corrosion potential and forming a metal passivation layer.^[^ ^170^ ^]^ PEDOT was coated onto steel surfaces to decrease the corrosion rate by shifting the corrosion potential to a more positive value. The steel surface was treated with an adhesive promotor, which binds itself covalently to the steel substrate and act as a self‐assembled monolayer which transform itself into the first layer of polythiophene to significantly improve the coating stability.^[^ ^160^ ^]^

### 3.7 Summary: Benefits of Organic Electrode Coatings

Electroconductive organic coatings have the potential to deliver electrical, physical/mechanical, chemical/electrochemical and biological benefits to metallic neuromodulation electrodes. Modifying stimulation electrodes with organic coatings offers significant electrical benefits, including reduced interfacial impedance, increased CSC and improved charge transfer mechanisms. Organic coatings may also provide intermediate modulus solutions to reduce mechanical mismatch between the very high modulus metals with very low modulus tissues. Another area where organic coating may add significant value is to biological performance, including the capacity of organic polymers to deliver therapeutics. Finally, although little studied in neuromodulation applications, organic coatings may provide protection from electrochemical corrosion and metal deterioration. This is an area that may be of significant benefit in such applications in an era where there is a growing need for much smaller electrodes.

Coatings may offer effective solutions to mitigate electrode corrosion. However, the demands on such coatings are extreme. Coating materials require intrinsically high thermal and chemical stability and continuous, defect‐free structures to be able to physically separate the metal surface from surrounding electrolytes. Such structures also need to prevent the diffusion of oxygen and other atoms adsorbing to the electrode surface and exhibit strong confinement effects on metal chemistry underlying the coating to reduce corrosion and dissolution and impede progression of corrosive reactions.^[^ ^172^, ^173^, ^174^, ^175^, ^176^, ^177^ ^]^ Additionally, coating materials require high electrical conductivity and should stabilize the passivation layer formed on the metal thus preventing further corrosion.^[^ ^155^, ^156^ ^]^ Such materials, especially, electroconductive polymers, offers the potential to shift the reaction site of oxygen reduction, identified as one of the key reactions causing metal dissolution, from metal‐polymer interface to the polymer‐electrolyte interface, thus limiting the reduction products interrupting metal surface.^[^ ^97^, ^160^ ^]^

In order to realize these benefits, the demands relating to device electrical and biological performance, including assuring device stability, must be met. Coatings need be stable, with strong adhesion to the underlying metal substrate, and approaches for evaluating coating quality, in particular measuring coating adhesion on ultra‐small electrodes must be simple and accurate.

## 4 Organic Coatings for Bioelectronics: Challenges

In the context of this review, there are several key challenges that must be addressed in developing organic conductive coatings for neuromodulation. First, robust bonding between organic coatings and metal substrates is crucial for the long‐term stability and performance of the neurostimulating devices. Additionally, it is important to understand the mechanisms of adhesion to translate this into techniques for improving and measuring coating adhesion.

### 4.1 Stability and Coating Adhesion

Achieving exceptional bonding strength between polymers and metals is challenging due to their distinct physicochemical differences. If the coating delaminates, the superior electrical, mechanical, and biological properties of the electrode would be compromised, potentially releasing coating material debris into surrounding tissue and causing reduced effectiveness or device failure.

Adhesion between dissimilar materials is a very complex and interdisciplinary subject that implies knowledge of surface chemistry, fracture mechanics, material science, rheology, and more.^[^ ^178^ ^]^ Although the study of adhesion mechanism dates back to the 1930s^[^ ^180^ ^]^ and has been accompanied by extensive research carried out since then with the aid of characterization technologies and computer simulation, describing adhesion mechanisms remains challenging due to the evolving understanding and complexity of the topic.^[^ ^178^, ^180^, ^181^, ^182^ ^]^

#### 4.1.1 Mechanism of Adhesion

While there are several proposed adhesion mechanisms described in the literature, it should be noted that there is some overlap between these mechanisms.^[^ ^181^, ^183^, ^184^, ^185^ ^]^ An overview of the four major adhesion mechanisms recognized is presented and discussed here. These include the electrical or electrostatic model, the diffusion model, the mechanical interlocking model, and the adsorption theory. Notably, the adsorption theory is now considered one of the most significant mechanisms through which adhesion is achieved.^[^ ^183^ ^]^ This theory encompasses several models, thermodynamic adsorption or wetting model, rheological model, chemical adhesion, and the weak boundary layer, which are occasionally referred to as separate mechanisms in literature.

##### Electrical or Electrostatic Theory

**Figure 6:**
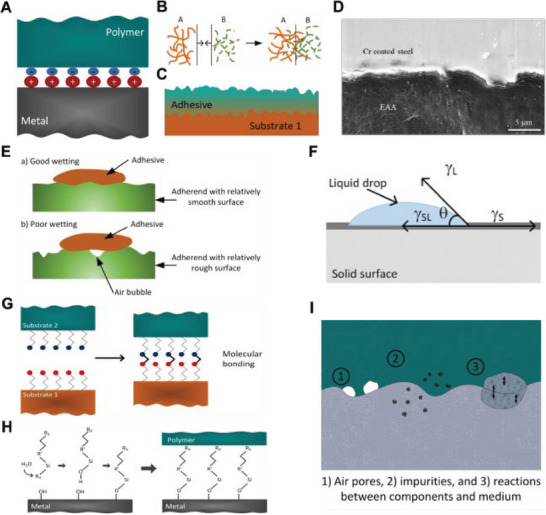
A) Schematic diagram of the electrical double layer formed at a polymer‐metal interface. B) Interdiffusion of macromolecules A and B from the superficial layer across the interface. Reproduced with permission,^[^ ^184^ ^]^ Copyright 1995, Wiley‐VCH. C) Illustration of mechanical interlocking between adhesive and substrates. Reproduced with permission.^[^ ^178^ ^]^ Copyright 2009, Elsevier. D) SEM image of typical interfacial morphology of Cr‐coated steel and EAA. The interface was rugged with concave and convex structures, indicating the formation of interlocking. Reproduced with permission.^[^ ^181^ ^]^ Copyright 2022, American Chemical Society. E) An example of good and poor wetting by an adhesive spreading across a surface. Reproduced with permission.^[^ ^185^ ^]^ Copyright 2014, William Andrew Publishing. F) Contact angle or wetting angle (θ) established at the intersection of solid‐liquid interface where a liquid droplet along with its vapor is at rest on a solid surface, θ describes the wettability of a surface by adhesive. Reproduced with permission.^[^ ^183^ ^]^ Copyright 2012, Elsevier. G) Schematic of the molecular bonding between substrates. Reproduced with permission.^[^ ^178^ ^]^ Copyright 2009, Elsevier. H) Schematic of silane‐modified interface: the hydrolysable alkoxy group (R_3_) forms intermediate silanol group (Si‐OH), which reacts with hydroxyl group on metallic surface, forming covalent siloxane bonds (M‐O‐Si) with metal substrate. The organofunctional group (R_x_) on the other end reacts and binds with polymer. Reproduced with permission.^[^ ^202^ ^]^ Copyright 1991, Springer. I) Model of weak boundary layer containing air pores, impurities, and reactions between components and medium. Reproduced with permission.^[^ ^183^ ^]^ Copyright 2012, Elsevier

The electrical or electrostatic adhesion theory suggests that when an adhesive‐substrate system constitutes a configuration of electron acceptor‐donor pairing, electrons tend to transfer from the donor (e.g., metal) to the acceptor (e.g., polymer), resulting in the formation of an electrical double layer at the interface as depicted in **Figure** **6**A.^[^ ^184^ ^]^ This double layer comprises a layer of positive ions near one surface and a layer of negative ions near the other. This concept was initially introduced by Derjaguin and colleagues in 1948.^[^ ^186^, ^187^ ^]^ They postulated that materials possessing dissimilar electron band structures undergo electron transfer to maintain Fermi equilibrium. While applicable to incompatible materials like metals and polymers, this model is unlikely a significant contributor to interface adhesion strength and does not explain the impact of factors such as temperature and moisture on separation force.

##### Diffusion Theory

The diffusion theory proposes that intimate adhesion occurs as the result of interdiffusion of macromolecules from the superficial layer of two compatible materials. This diffusion takes place across the interface between the adhesive and adhered material (Figure 6B).^[^ ^184^, ^188^ ^]^ The theory is primarily applicable when both the adhesive and adherend are polymers with relatively long‐chain molecules capable of movement. Therefore, it is likely not relevant in the case of polymer‐metal interfaces.

##### Mechanical Interlocking Theory

The theory of mechanical interlocking was first proposed by McBain and Hopkins in 1925 as one of the first attempts to explain the science behind the adhesion at interface.^[^ ^179^ ^]^ This theory suggests that adhesion originates from the penetration of adhesive into pores, cavities, and other surface asperities on the adherend or substrate (Figure 6C).^[^ ^178^ ^]^ The strength of mechanical adhesion is directly related to the degree of irregularities such as roughness and porosity of the substrate. When a fluid‐state polymer is applied onto a metal surface, it infiltrates the macroscopic surface structures and forms an adhesive composite as the polymer material solidifies.^[^ ^189^, ^190^ ^]^ This phenomenon can be observed microscopically, as seen in the interfacial morphology of chromium‐coated steel and ethylene acrylic acid (Figure 6D), displaying concave and convex structures that indicate the formation of mechanical interlocking.^[^ ^181^ ^]^

The role and significance of interlocking in adhesion rely on surface irregularities and the theory cannot explain the adhesion phenomenon on perfectly smooth surfaces.^[^ ^185^ ^]^ Moreover, the theory does not consider any interatomic or intermolecular interactions between materials at microscopic scale. Therefore, the mechanical interlocking theory should be considered as a co‐factor in enhancement of adhesion strength.

##### Adsorption Theory

The adsorption theory proposes that adhesion results from interatomic and intermolecular forces that are established across adhesive‐substrate surface following their intimate contact. Introduced by Sharpe and Schonhorn,^[^ ^191^ ^]^ it describes a two‐stage process for adhesive interface formation. Initial adherence involves physical adsorption such as Van der Waals forces, attracting atoms on the material surface.^[^ ^182^, ^192^, ^193^ ^]^ Since Van der Waals forces are effective over a small range, surface atoms must be brought closely together for the force to operate. Chemical adsorption, involving molecular interactions, takes place after initial molecular contact has been established. This theory emphasizes that a strong adhesive joint requires increasing the real contact area between two materials. In practice, this involves a liquid‐phase material with low surface tension contacting a solid with high surface energy.^[^ ^191^ ^]^ The liquid component should spread spontaneously over and into the micro‐ and macro‐pores of the solid surface, maximizing contact area and increasing the rate to approach the equilibrium state. This spread ability‐adhesion criteria gives rise to the development of thermodynamic adsorption or wetting theory.

###### a) Thermodynamic Adsorption or Wetting Theory

The theory suggests that the occurrence of adhesion is only possible when there is intimate, continuous contact at the liquid‐solid interface, establishing wettability. For a liquid adhesive to wet the surface, the liquid should possess lower surface tension than the critical surface tension of the solid. Figure 6E illustrates an example of good and poor wetting by an adhesive spreading over an adherend. “Complete” wetting maximizes the contact area by filling all crevices on the substrate surface, whereas “incomplete” wetting generates surface defects, leading to reduced contact area.^[^ ^185^ ^]^ Young quantified wetting by considering an equilibrium state where a liquid droplet along with its vapor is at rest on a solid surface (Figure 6F).^[^ ^183^, ^184^ ^]^ The relationship between the surface energies of the solid‐liquid system is described as:^[^ ^194^ ^]^

(4) $$\gamma_{s}=\gamma_{sl}+\gamma_{l}cos\theta_{sl}$$

where γ_s_ is the surface energy of the solid substrate in contact with liquid vapor, γ_*l*_ is the surface energy (or surface tension) of the liquid droplet in contact with its vapor, γ_sl_ is the surface energy at solid‐liquid interface, and θ is the wetting or contact angle that describes the intersection of three interfaces. Provided that the surface energy is closely associated with the adhesion, the thermodynamic work of adhesion (W_sl_) defined by Dupré is expressed as:^[^ ^195^ ^]^

(5) $$W_{sl}=\gamma_{s}+\gamma_{l}-\gamma_{sl}$$

The contact angle of the liquid on the surface can be related to the work of adhesion, leading to:

(6) $$W_{sl}=\gamma_{l}\left(1+cos\theta_{sl}\right)$$

This equation is a useful measurement of the adhesion strength of a particular system given the surface energies and contact angle can be measured experimentally.

###### 4.1.1.1 b) Rheological Model

Adhesion strength is also highly influenced by the internal stress within the system. This stress arises from various factors during the formation of polymeric coating and interactions with the solid substrate.^[^ ^184^ ^]^ This leads to the establishment of rheological model that correlates failure energy (W) to the rheological characteristics of the adhesives, namely, their bulk properties as follow:^[^ ^196^ ^]^

(7) $$W=W_{o}\;f\left(V,T\right)$$

where W_o_ is Dupré’s work of adhesion, which only depends on the material surface properties as discussed earlier, while f(V, T) is a function of separation rate and temperature. This function accounts for energy dissipation during irreversible deformation of polymers as the point of failure propagates. This property is only dependent on the bulk properties of the adhesives.^[^ ^197^ ^]^ In certain studies, a molecular dissipation factor was introduced to the relation to account for irreversible breakage of bonds between crosslinks in polymer chains. This factor is related to the number of C‐C bonds between two crosslinkers and, consequently, the molecular weight.^[^ ^198^ ^]^ This theory effectively explains the variation in adhesive and cohesive properties of a polymer with their degree of crosslinking. It also differentiates the contribution of material surface and bulk characteristics to adhesion, complimenting the thermodynamic adsorption theory.

###### c) Chemical Adhesion

Chemical adhesion frequently takes place on polymer‐metal interfaces, involving a transition from crystalline lattice metal structure to molecular polymeric structure. The theory suggests that adhesion is attributed to the interfacial forces between two dissimilar contacting materials.^[^ ^192^ ^]^ This entails the intermolecular forces such as dipole‐dipole interaction, Van der Waals force, hydrogen bonding, and chemical bonding such as ionic, metallic, and covalent bonds (Figure 6G).^[^ ^178^ ^]^ It is widely accepted that the formation of chemical bonding greatly enhances adhesion strength at the interface, with bonding ranging from 40–400 kJ mol^−1^, significantly surpassing the bonding energy of intermolecular forces of ≈2–40 kJ mol^−1^, making it the primary force of adhesion.^[^ ^183^, ^199^ ^]^

Chemical bonds formed at the interface are the result of charge transfer from metal to polymer. For example, the presence of C‐O‐Metal complex was observed on various metal surface.^[^ ^181^, ^200^ ^]^ Chemically grafting a polymerizable organic molecule via terminal carbon or nitrogen atoms onto the metal substrate enables formation of covalent bonds at the polymer‐metal interface create.^[^ ^201^ ^]^ Additionally, chemical bond occurs in the bridging mechanism involving the use of coupling agent such as silanes (Si).^[^ ^189^ ^]^ Silane coupling agents with the general structure of R_3_Si(CH_2_)_n_R_x_, (R for substrate‐reactive hydrolysable group, R_x_ for polymer‐reactive organofunctional group), create highly crosslinked covalent bonds at the interfacial region as demonstrated in Figure 6H.^[^ ^183^, ^202^ ^]^ These agents improve hydrolytic stability of the joint composite by maintaining equilibrium between the silane‐modified metal and polymer surfaces.

###### d) Weak Boundary Layer Theory

The weak boundary layer (WBL) theory, proposed by Bikerman,^[^ ^203^ ^]^ reveals the existence of a finite layer with properties differing from the bulk material that forms it. Stress relaxation and crack propagation in the WBL are different from those in conventional interfaces, thus greatly impacting the overall material performance. The WBL can be caused by air when the substrate is poorly wetted by the polymer or contaminates, which could be impurities present in either the substrate and adhesive, or products of reactions between the material and environment (Figure 6I).^[^ ^183^ ^]^ The theory suggests that for a satisfactory performance of adhesion, the WBL should be eliminated.

#### Summary of Adhesion Mechanisms

Adhesion mechanisms are complex with multiple interconnected factors that are likely to contribute to the formation of adhesive joints between dissimilar materials. Among the theories proposed, mechanical interlocking, thermodynamic adsorption, rheology, chemical bonding, and weak boundary layer are likely the most relevant theories concerning the adhesion of organic coatings on metal electrode surfaces. However, the complexity of biological environments can significantly impact coating stability.

#### 4.1.2 Coating Stability in Vitro and In Vivo

Despite the electrical, mechanical, and biological benefits of organic coatings on the performance of stimulating electrodes, coating robustness is the key factor that determines the long‐term safe operation of the devices. Organic coating may fail through deterioration of the polymer, or they may delaminate completely from the metal surface.

Traditional PEDOT:PSS coatings exhibit low interfacial toughness (10 J m^−2^), leading to cracking and delamination after multiple charging‐discharging cycles.^[^ ^204^ ^]^ CP films are often brittle and the incorporation of dopants into the polymer structure can further exacerbate this effect.^[^ ^101^ ^]^ The doping and dedoping process upon the application of external potential results in a flux of dopant ions in or out of the film, disrupting the stable polymer backbone and cause substantial volume change to the CP film.^[^ ^88^, ^205^, ^206^ ^]^ Such dimensional change can be extremely large and influence the mechanical properties of CP. Furthermore, the oxidative state of dopant was suggested to contribute to the degradation of the polymer film through overoxidation. PEDOT doped with heparin and PSS completely lost electroactivity within 22 days when exposed to hydrogen peroxide (H_2_O_2_) solution at 37 °C due to oxidation attack breaking double bonds in the polymer backbone.^[^ ^81^ ^]^ CP formed in a reducing medium, such as oxalic acid, demonstrated better stability compared to those formed in electrolytes containing perchloride and nitrate ions.^[^ ^205^ ^]^

The stability of CP can be assessed electrochemically through a variety of electrical approaches. Electrochemical impedance spectrometry (EIS) provides information on the charge transfer characteristics of the electrode‐CP‐electrolyte interface and cyclic voltammetry (CV) can determine the charge‐carrying capacity of CP film which can infer adhesion and delamination of CP coatings. The stability of PEDOT coatings on various metal substrates including Pt,^[^ ^107^ ^]^ Au,^[^ ^86^ ^]^ and indium tin oxide^[^ ^81^, ^106^ ^]^ was assessed under various conditions relevant for the application in neural stimulating devices. PEDOT:PSS on gold substrate retained its original electroactivity after 400 CV cycles,^[^ ^86^ ^]^ while PEDOT:PSS on Pt samples showed minor cracks and delamination after two weeks of biphasic stimulation, accompanied by a shift in redox peak, and abrupt increase in impedance.^[^ ^107^ ^]^

When CPs were tested in physiological solution using fetal bovine serum (FBS), PEDOT:PSS exhibited an impedance increase of less than 10% during incubation.^[^ ^207^ ^]^ However, when subjected to a two‐week stimulation, PEDOT experienced 42.8% and 13.6% reduction in electroactivity when doped with pTS and LiClO_4_ respectively.^[^ ^208^ ^]^ These reductions in electroactivity were associated with polymer chain rearrangement and mobile dopants diffusing out of the polymer during charging and discharging process.

Most studies on the stability of CP coatings have been conducted in a simulated in‐vitro environment for short periods of time. Limited research evaluating chronic in‐vivo performance of coatings has been reported. In vivo evaluation of PEDOT‐based coatings revealed an initial rapid increase in impedance of coated electrodes upon implantation with a plateau evident over time. This impedance increase was associated with formation of fibrous tissue rather than coating delamination.^[^ ^209^, ^210^ ^]^ Equivalent circuit analysis of the interface revealed that the enhancement of surface area by coating was diminished, barrier composed of insulation begins to dominate the impedance spectrum. SEM image confirmed this with the presence of dense membranous substances enveloping the coating pores. It was suggested that the presence of tissue surrounding the electrode may prevent coating from detachment as observed in vitro.

Decomposition or delamination of coatings induced by repetitive potential cycling is likely to be less severe in vivo than in PBS, where anions diffusion and ions exchange is unimpeded compared to within tissue.^[^ ^211^ ^]^ It should be noted that although no significant delamination was reported, the effectiveness of coatings is limited by the significant impedance increase after chronic implantation.^[^ ^89^, ^95^, ^212^ ^]^ It is therefore essential to perform long‐term in‐vivo studies for better understanding of the impact of biological environment on the long‐term stable performance of the organic coatings.^[^ ^101^ ^]^ A 12‐week chronic implantation study was performed to assess the stability of PEDOT/CNT‐coated electrodes. Despite demonstrating superior electrical performance, coating material degradation was observed, indicating the need for further improvement in material coating adhesion.^[^ ^213^ ^]^ Overall, weak electrochemical and mechanical stability of CP coatings poses a significant challenge for their use in chronic stimulating devices.

**Figure 7:**
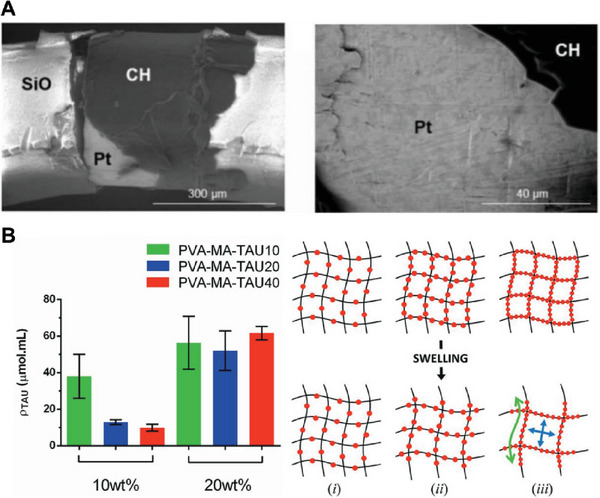
A) Effect of stimulation on surface structure of CH‐coated Pt electrodes: CH‐coated electrode (left), and region of CH loss exposing the underlying Pt surface (right). Reproduced with permission.^[^ ^121^ ^]^ Copyright 2020, IOP Publishing. B) CH produced from different anionic dopant densities from 10 wt.% and 20 wt.% PVA‐MA precursor solution (left), and the swelling behavior of gels with low i), intermediate ii), and high (iii) degrees of taurine substitution. Taurine was shown as red dots. Reproduced with permission.^[^ ^124^ ^]^ Copyright 2017, Wiley‐VCH.

Chronically stimulated CH coated electrodes have shown various degrees of coating loss and release of particulate polymeric material to the adjacent tissue (**Figure** **7**A).^[^ ^121^ ^]^ Swelling of hydrogel coating upon implantation can cause substantial dimensional change, potentially compromising the mechanical stability.^[^ ^130^ ^]^ The presence of CP content in the hydrogel is expected to further influence the dehydration/rehydration behavior of the gels.^[^ ^214^ ^]^

The introduction of taurine groups was found to increase the swelling due to increased electrostatic repulsion between anionic sulfonate groups, leading to increased mesh size (Figure 7B).^[^ ^124^ ^]^ While the high‐density pathway of taurine dopant along the hydrogel polymer backbone ensures superior electrical properties, it is crucial to manage the swelling behavior of coatings. A low swelling CH blend of PVA‐MA and PVA‐Tau demonstrated comparable electrochemical performance to pure PVA‐Tau formed CH.^[^ ^130^ ^]^ However, high methacrylate functionalization can impact on the hydrogel network permeability, which in turn can result in suboptimal deposition of CP. Therefore, optimal growth of CP within hydrogel requires a balance between electrical and mechanical properties for formation of cohesive CHs.

During long‐term implantation and stimulation, repetitive charging and discharging are likely to cause local stress within CH coatings, gradually forming cracks, leading to interfacial delamination due to the fragile CH‐electrode interface. As discussed previously, the expansion and shrinkage of CP under repetitive potential cycling can cause periodic changes in thickness and modulus of the layer, resulting in accumulation of shear stress around the coating edge.^[^ ^130^ ^]^ Cracks form when applied stress exceeds the interfacial toughness of the interface. Despite the advancements in developing interpenetrating CH coatings with remarkable electrical and biological properties, their inherent swelling nature and weak bonding to metal substrates pose challenges for coating cohesion and adhesion and integration into chronic implantable devices.

Enhancing the interfacial toughness is crucial for effectively improving the coating adhesion and long‐term stability of CH coated electrodes. This can be achieved through increasing the intrinsic work of adhesion and introducing mechanisms for mechanical energy dissipation during hydrogel deformation. Chemically anchoring the hydrogel polymeric network to metal substrates can significantly increase the intrinsic work of adhesion at the hydrogel‐metal interface.^[^ ^215^ ^]^ Employing long‐chain polymer or large crosslinkers with high functionality can also increase the adhesion fatigue threshold.^[^ ^216^ ^]^ Those long‐chain networks can maintain the elasticity and cohesiveness of the hydrogels even if short chains are ruptured.

Physical crosslinking that can be recovered upon decrosslinking can be incorporated into hydrogel networks to dissipate mechanical energy during hydrogel deformation, further contributing to increasing interfacial toughness.^[^ ^217^ ^]^ A tough hydrogel, fabricated by using long‐chain polymer of polyacrylamide (PAAm) or polyethylene glycol diacrylate (PEGDA), interpenetrated with reversible crosslinked network of alginate, has been covalently attached to silane modified surfaces. This hydrogel exhibited remarkable interfacial toughness, measuring over 1000 J m^−2^ on various solid substrates.^[^ ^215^ ^]^

While the function and stability of organic coatings has been studied in biological environments, systematic approaches for understanding the relative contribution of different adhesion mechanisms are lacking. There is a need to better understand adhesion mechanisms in physiological conditions and, equally important, the stability of bonds formed needs to be considered under relevant environments for the likely duration of operation.

#### 4.1.3 Improving Coating Stability

Improving adhesion is crucial for enhancing the properties of coatings. The weak and unstable adhesion of organic coatings to neural electrodes greatly limits their application in establishing a reliable neural interface. In light of this challenge, various methods have been studied to enhance the polymer‐metal adhesion, with some approaches reporting promising results. An overview of techniques explored in literature to improve organic coating adhesion is reported, while also considering the underlying adhesion mechanisms.

**Figure 8:**
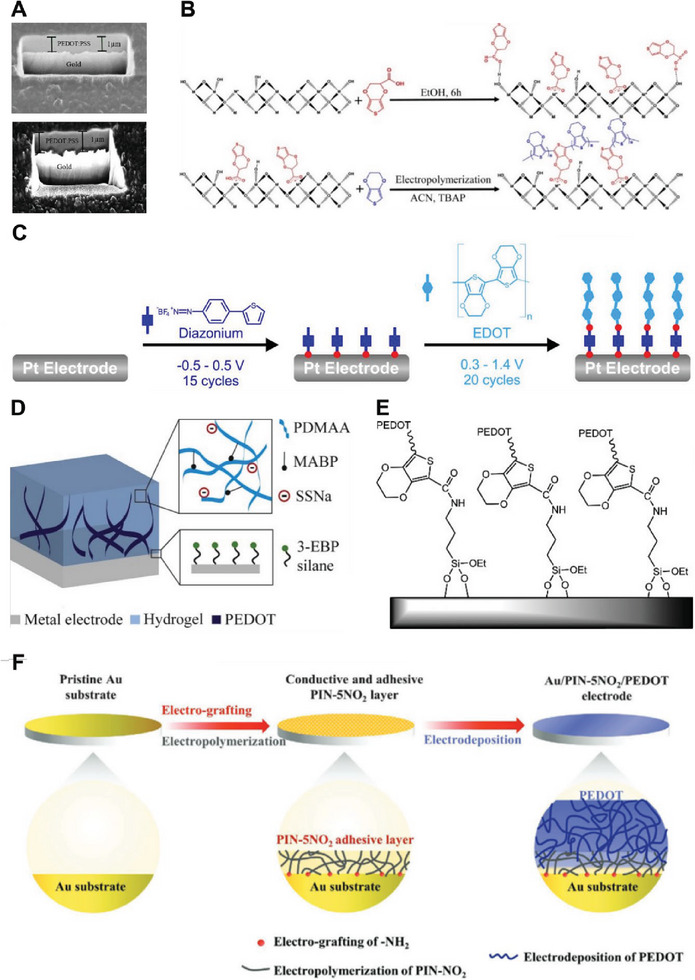
A) SEM image of iodine etched electrodes coated with PEDOT without (top) stimulation and with (bottom) stimulation. The vertical cut through clearly shows that PEDOT infiltrates into the pores of Au surface, establishing mechanical anchoring. Reproduced with permission.^[^ ^218^ ^]^ Copyright 2018, Elsevier. B) Chemisorption of EDOT‐acid (red) into ITO structure (black), followed by electrodeposition of PEDOT (blue). Reproduced with permission.^[^ ^80^ ^]^ Copyright 2015, American Chemical Society. C) Schematic illustrating the two‐step process of covalent attachment of PEDOT on Pt electrode through grafting of diazonium salt, followed by polymerization of PEDOT initiated by the phenylthiophene layer. Reproduced with permission.^[^ ^79^ ^]^ Copyright 2018, The Electrochemical Society. D) Covalently bound CH consisted of PDMAA backbone, MABP crosslinker, and SSNa as counterions during polymerization of PEDOT. The covalent attachment is achieved through 3‐EBP silane. Reproduced with permission.^[^ ^125^ ^]^ Copyright 2017, Elsevier. E) Schematic illustration of PEDOT covalently grown on EDOT‐aminopropyltriethoxysilane‐modified conductive substrate. Reproduced with permission.^[^ ^226^ ^]^ Copyright 2014, American Chemical Society. F) Electrografting of a conductive PIN‐NO_2_ adhesive layer, followed by electrodeposition of PEDOT. Reproduced with permission.^[^ ^227^ ^]^ Copyright 2021, Wiley‐VCH.

Surface roughening procedures such as etching, abrasion, and laser patterning, are frequently used on metals to improve polymer‐metal adhesion by facilitating mechanical interlocking.^[^ ^185^ ^]^ These techniques create more surface area and anchoring points for coating materials to conform to the substrate, thus increasing adhesion strength. Textured electrode surfaces can be achieved through surface etching, introducing nanostructures, and laser patterning. For instance, selective etching of gold electrodes with iodine forms pores on the electrode surface that enhance PEDOT:PSS anchoring during polymerization (**Figure** **8**A). Improved stability is demonstrated through in‐vitro stimulation and sonication tests, showing minimal changes in EIS results after 604 million stimulation pulses and surviving aggressive mechanical agitation throughout the test period.^[^ ^218^ ^]^

Introduction of nanostructured Pt or IrO_x_ layer through electrodeposition and Au nanorod via dealloying produces nanostructured topographies supporting the anchoring CP coatings.^[^ ^219^, ^220^ ^]^ PEDOT adhesion on IrO_x_ endures CV stressing with no delamination, while nanostructured Pt exhibits small cracks. Comparatively, unmodified Pt experience PEDOT delaminates in large pieces shortly after the stress testing. Au nanorods enabled a 10‐fold increase in the number of CV cycles endured by PEDOT coating compared to unmodified gold electrodes. Laser roughened Pt electrodes significantly improve the passive stability of PEDOT coating evaluated at elevated temperature, and extend the chronic stimulation lifetime by delaying the failure of coating by 500 million pulses compared on smooth Pt.^[^ ^221^ ^]^

Attaining optimal adhesion relies on intimate contact between adhesive and substrate. This can be promoted by enhancing substate wettability by adhesive, reducing surface tension of the polymer solution, and increasing the surface energy of the metal electrode. It is known that the strong intermolecular interactions like hydrogen bonding yield high surface tension in liquids. Appropriate substitution of fluorine or fluorinated side chains for hydrogens in hydrogen‐containing organic materials for example, can result in lower surface tension.^[^ ^191^ ^]^

Surface treatment such as using plasma activation consisting of bombarding the substate surface with activated atoms of gases, can increase the surface energy and reduce the contact angle of the substrate,^[^ ^184^, ^185^ ^]^ improving the wetting of the substrate by the adhesive. Plasma treatment is an effective way of modifying surface properties without changing the overall bulk properties of the material.^[^ ^185^, ^222^ ^]^ Functional groups such as carboxyl, hydroxyl, and carboxylic can be formed on target surface through oxygen‐ and carbon‐containing plasma to increase surface wetting and improve adhesion.^[^ ^178^ ^]^ Nitrogen plasma treatment is also recorded to increase adhesion where surface polarity was seen increased following the treatment, reflecting a higher work of adhesion between the metal and polymers.^[^ ^223^ ^]^

Chemical adsorption, including intermolecular forces and chemical bonding are expected to greatly enhance the coating‐substrate adhesion. Promoting chemisorption by forming hydrogen bonds between organic molecules and activated metal surface,^[^ ^80^, ^224^ ^]^ electrografting a polymerizable organic molecules to form metal‐organic covalent bonds,^[^ ^225^ ^]^ and employing coupling agents that capable of forming covalent bonds at both polymer and metal ends,^[^ ^226^ ^]^ are all expected to significantly improve adhesion. For instance, indium tin oxide (ITO) coated glass slides that were UV activated and dipped into a carboxylic modified EDOT acid solution caused formation of a densely packed monolayer through chemisorption of carboxylic groups on the treated ITO surface. PEDOT was subsequently electropolymerized onto the modified substrate, forming a highly stable coating (Figure 8B).^[^ ^80^ ^]^

Electrografting methods have been developed to improve film adhesion by creating covalent bonds between organic molecules and conductive solid substrates, during which organic molecules are oxidized or reduced, followed by formation of organometallic complexes.^[^ ^201^ ^]^ Pt‐Ir electrodes were modified by grafting (4‐thien‐2‐yl) diazonium salt through electrochemical reduction. PEDOT was then electropolymerized on the grafted phenylthiophene layer (Figure 8C).^[^ ^79^ ^]^ The diazonium‐based anchoring significantly improved coating adhesion, maintaining their initial morphologies after 1000 cycles of CV scans, whereas physically adsorbed PEDOT film delaminates after 40 cycles. A similar strategy involved electrografting an amine‐functionalized EDOT derivative onto a range of conductive substrates to form a well‐adherent Poly (2,3‐dihydrothieno[3,4‐b][1,4]dioxin‐2‐yl)methanamine (P(EDOT‐NH_2_)) layer. This is followed by deposition of PEDOT, resulting in comparable electroactivity to pristine PEDOT coating, but greatly enhanced adhesion.^[^ ^225^ ^]^

Adhesive promotors, such as silane coupling agents, are used to form an intermediary layer, to improve the adhesion strength between two dissimilar materials.^[^ ^202^ ^]^ By employing 3‐(trimethoxysilyl) propyl methacrylate (TMSPMA) to a conducting ITO substrate, permeable methacrylate moieties are formed on the substrate surface. Subsequent chemical grafting of poly(styrene sulfonate‐co‐4‐vinyl pyridine) (Poly (SS‐4VP)) through free radical copolymerization, followed by electrochemical deposition of EDOT, yields an interpenetrating CH network covalently anchored to the metallic substrate.^[^ ^204^ ^]^ The synergic effect of covalently anchoring of Poly (SS‐4VP) and chemical crosslinking of PEDOT within the hydrogel network significantly improves the long‐term stability of coating under stimulation, maintaining 91% of its original electroactivity after 10000 charging‐discharging cycles.

In a similar approach, IrO_x_ surface functionalized with 4‐(3‐triethoxysilyl) propoxybenzophenone (3‐EBP) silane was used to form a benzophenone group on IrO_x_ surface. Subsequent electrodeposition of PEDOT within P(DMAA‐co‐5%MABP‐co‐2,5%SSNa) (PDMAAp) hydrogel leads to permanent integration of CP into the hydrogel scaffold, significantly improving interfacial adhesion (Figure 8D).^[^ ^125^ ^]^

The EDOT monomer can also be modified to incorporate an alkoxysilane end for directly grafting onto a conductive substrate. The irreversible oxidation of amine groups in the presence of unsubstituted EDOT monomers triggers the cationic polymerization of EDOT, resulting in the growth of PEDOT chains directly linked to the conductive substrate (Figure 8E).^[^ ^226^ ^]^ The resulting film exhibited strong adherence to the substrate compared to conventional PEDOT film, as evidenced by adhesion tape and sonication tests.

However, a key limitation associated with an adhesive anchoring layer is its poor conductivity, potentially compromising the electrical performance of neural electrodes. One work around involves introducing a conductive interfacial layer such as poly(5‐nitroindole) (PIN‐5NO_2_), which possesses excellent electrochemical properties. During the redox process of 5‐nitroindole, partial reduction of nitro group in PIN‐5NO_2_ yielded an amino group that can be further electro‐grafted onto Au substrates via covalent bonds (Figure 8F). With a PIN‐5NO_2_ thin film as the adhesive interfacial layer, subsequent electrodeposition of PEDOT demonstrates excellent adhesion to Au substrates, showing no significant performance decline after 30 min of ultrasonication and 2000 CV cycles.^[^ ^227^ ^]^

From a rheological perspective, enhancing interfacial adhesion strength involves increasing the intrinsic fracture toughness of the bulk material and implementing energy‐dissipation mechanism into material during deformation.^[^ ^216^ ^]^ Composite coatings of PEDOT and CNTs exhibit higher stability compared to smooth PEDOT counterpart due to their interdigitated network structure. The mechanically strong CNTs are evenly distributed across the polymer film, increasing the intrinsic fracture toughness of the composite, preventing the film from delamination due to expansion and shrinkage of CP film. The porous structure of composites can also effectively reduce internal stress buildup during CP actuation.^[^ ^63^, ^65^, ^83^, ^84^, ^228^, ^229^ ^]^ Hydrogels tailored with reversible crosslinkers and interpenetrating long‐chain network display exceptional interfacial roughness over 1000 J m^−2^, far exceeding the 10 J/m^−2^ of conventional polymer coatings.^[^ ^215^, ^217^ ^]^

Optimal adhesion entails eliminating the weakest link of the interfacial joint, the WBL. This can be achieved through proper surface treatment that removes contaminants, including cleaning through physical, mechanical, or chemical means, and plasma cleaning for thorough surface modification.^[^ ^185^ ^]^ Contaminants such as residual moisture, organic substances, greases, oxide layers and dirt that accumulate on the metal or polymer surface can lead to the formation of WBL at the metal‐polymer interface, hindering direct interaction between two materials. Various preparation methods are employed to clean metal surfaces effectively. Solvents like acetone, isopropyl alcohol, or chloroform are applied.^[^ ^185^, ^230^ ^]^ Plasma cleaning, degassing with hot air or steam, and UV zone treatment further aid in comprehensive metal surface preparation.^[^ ^231^ ^]^

For polymer‐coated neural stimulating electrodes used in vivo, repetitive charging and discharging of the conductive coating is known to induce internal stress that may lead to imperfections or microcracks, enabling rapid fluid ingress into the interface. Water molecules are excellent hydrogen bonds donor and acceptor, they can readily hydrolyze hydrogen or even covalent bonds formed at polymer‐metal interface. In a complex in vivo environment, factors like temperature, moisture, and biological factor can synergistically act on the surface, reducing adhesion strength.^[^ ^185^ ^]^ Therefore, it is important to evaluate the adhesive system in simulated environments that represent the actual operation conditions that coatings will encounter.

#### 4.1.4 Techniques for Evaluating Coating Stability

The development of novel coating technologies for use in neuroprosthetic devices requires extensive preclinical testing to demonstrate reliability. Although chronic in vivo testing can be useful, the time and cost associated with animal studies underscore the importance of developing validated in vitro methodologies that allow for more expedient testing of novel electrode materials.^[^ ^232^ ^]^ Evaluating coating adhesion is an essential step in the assessment of the long‐term stability of coated electrodes.

Various theories have been proposed on failure modes occurring at the polymer‐metal interface that contribute to declines in performance.^[^ ^233^ ^]^ **Table** **1** outlines the range of direct testing methods, including adhesion‐by‐tape test, peeling test, scratch test, and lap shear test, as well as indirect methods such as sonication, accelerated aging, cyclic voltammetry, and biphasic stimulation. These methods have been used to measure adhesion either qualitatively or quantitively, however many of them have not been standardized and results may therefore vary considerably across users.

**Table 1:** Overview of testing methods used to evaluate adhesion of organic coatings to metal substrates.

Test	Explanation	Reference
Direct adhesion testing		
Adhesion by tape test	To mechanically assess coating adhesion. Referring to American Society for Testing and Materials standard – ASTM D3359 as a guideline, an incision of “X” is cut into the coating to expose the underlying metal. Adhesive tape is pressed over the incision for a defined time, then removed. The site is examined under microscopy (e.g., SEM) to assess the loss of coating.	[123, 221, 234, 235]
Peeling test	To derive interfacial toughness from the plateau force in a force‐displacement curve. Referring to ASTMD286, the prepared samples are tested in a standard 90^o^ peeling test set up. The sample is peeled at a constant peeling rate, the plateau value of the force‐displacement curve gives the adhesion energy.	[236, 237]
Pull‐off test	To determine the maximum perpendicular tensile strength an adhesive junction can sustain before failure (ASTM D4541).	[238, 239]
Scratch test	To assess coating failure mode. The coating surface is scratched at a fixed rate with a diamond stylus under increasing normal load until a critical value is reached at which the coating failure occurs. The scratches are examined under microscopy for evidence of failure modes.	[234]
Lap sheer test	To assess the interfacial shear strength between two substrates. An assembly fabricated by sandwiching the coating material between two targeted adherends, and pulling both ends of the assembly until adhesion or cohesion failure occurs. The maximum strength during the process is taken as the interfacial strength.	[240, 241]
Indirect adhesion testing		
Ultrasonication	To determine the duration the coating can withstand before delaminating. The coated electrode is sonicated in water for various lengths of time, and the degree of damage is assessed by microscope image or electrochemical properties.	[218, 225, 242]
Soaking and/or accelerated aging	To passively accelerate the degradation of coating in a controlled environment. Referring tointernational organization for standardization (ISO) 10993‐13 as a guideline, the coated electrode is soaked in PBS or saline that represents the in vivo environment. The surface condition and electrical properties of the electrode is assessed after the aging process.	[219, 221, 234, 235, 243]
Cyclic voltammetry (CV)	To assess how many charge‐discharge cycles the coating can endure before delamination. CV scans can be employed to induce stress to CP or CH coatings. The electrical and optical characteristics of the coating can be used as measures of adhesion quality.	[219, 233]
Biphasic stimulation	To examine coating stability under biphasic stimulation. Subjecting coated electrodes to high charge density stimulation, electrical and optical characterization can be used to determine changes in biphasic response and morphology of the coating over the time course of the study.	[127, 128, 221, 233]

While direct tests of mechanical properties are often used to quantify the adhesion strengths at the joint interface, these tests encounter several issues when applied to neural stimulating electrodes. One significant challenge is the small size and geometry of neural electrodes, making it difficult to conduct these mechanical tests effectively. The intricate geometries and curvatures of neural electrodes can complicate testing procedures, leading to inaccurate results and potential damage to the delicate structures.

Notably, direct adhesion tests may also fail to replicate actual failure modes occurring to the coating in the in‐vivo environment, where factors such as physiological interactions, dynamic movement, and long‐term stimulation induced stresses contribute to the adhesion issues. The use of direct adhesion test as a quantitative measurement of adhesion should ensure that failure events represent the loss of adhesion.^[^ ^244^ ^]^ For example, it was found that a range of non‐adhesive failure such as cohesive failure within the coating tends to occur when a scratch test is conducted on a brittle coating material such as CP coated on a ductile substrate like a Pt electrode.^[^ ^245^ ^]^ Therefore, careful consideration is necessary if the test is intended to evaluate coating‐substrate adhesion, where an adhesive failure should be induced with no interference from other failures.

Indirect testing methods offer several advantages for assessing coating stability on neural electrodes. Although these techniques do not provide quantitative adhesion measurements, they can be tailored to simulate in vivo environments, reproducing the degradation of coating materials. Ultrasonication, employed to mechanically agitate testing solution is based on the premise that if the coating withstands such strong vibrational force, it can also endure other mechanical stresses and fluid dynamics encountered in vivo.^[^ ^218^, ^225^, ^246^ ^]^ Ultrasonication effectively removes loosely bonded coating particles, thereby demonstrating the mechanical stability.

Soaking medical devices in saline at an elevated temperature has been frequently applied to accelerate material aging.^[^ ^232^, ^234^, ^247^ ^]^ This acceleration process is assumed to follow first‐order kinetics as described by the Arrhenius equation.^[^ ^248^ ^]^ This equation states that the rate of chemical reactions occurring at 37 °C is increased by a factor of 2, (Q_10_) for every 10 °C temperature rise.^[^ ^249^, ^250^ ^]^ The “aging factor” (AAF) can be estimated from the equation:

(8) $$AAF=Q_{10}^{\frac{T_{AA}-T_{RT}}{10}}$$

The accelerated aging temperature (T_AA_) should be below the transition temperature of the material of interest.^[^ ^251^ ^]^

A comprehensive analysis of the stability should also include the expected interactions between coating material and ion species present in the human body. Polymer coatings are susceptible to hydrolytic and oxidative attack to specific bonds in their structure.^[^ ^233^ ^]^ CPs and ionic dopants are known to undergo ion exchange with surrounding electrolyte, making them prone to degradation in aqueous media. Potential immune responses release digestive enzymes and reactive oxygen species that may react with polymer chains, breaking double bonds in the conjugated structure.^[^ ^232^ ^]^ Adding hydrogen peroxide into the testing solution acts to over‐oxidize the sulfur atom in CP, mimicking its deterioration in vivo.^[^ ^243^, ^252^, ^253^ ^]^

Additional factors such as biofouling and protein adsorption as well as the anisotropic properties of tissues should also be considered when performing an accelerated aging test.^[^ ^233^ ^]^ For electrochemical stress, repeated potential cycling of coated electrodes enables investigation of degree of reversibility of reactions associated with charge injection and reflects surface changes in peak shifts on the CV curve.^[^ ^219^ ^]^ This method serves as a measure for the electroactivity loss of a coating material and provides insight into the stability of polymer backbone and mobility of dopant ions. CPs undergo substantial volumetric changes and swelling during repetitive charging‐discharging cycles. This expansion induces forces at the adhesion interface, resulting in the buildup of internal stress and coating delamination. It is important to note that CV operates at a much longer time scale, ranging from seconds to tens of seconds, as opposed to the microseconds used in neural stimulating devices. This makes it a measure of the most extreme electrochemical conditions applicable to a coating material.^[^ ^128^, ^221^, ^242^ ^]^ High charge density stimulation of coated electrodes replicates volumetric changes in the coating material driven by the charge transfer process, mechanically challenge the bonding strength. The electrical performance such as voltage transient response, impedance, and CSC indirectly indicates adhesion quality.^[^ ^128^, ^221^, ^242^ ^]^

Further improvements in standardizing tests of coating stability are essential for evaluating neuromodulating devices, rather than focusing on laboratory testing of larger, less complex forms. Such tests should ideally be integrated into production lines or as post‐production quality control. Coatings must also be able to be manufactured efficiently, in a setting that meets commercial production requirements, and the following section briefly considers conductive coating manufacturing approaches.

### 4.2 Manufacturing Processes for Organic Coatings

The manufacturing methods of organic coatings include two main approaches: i) synthesis of CHs and ii) coating or synthesis of CHs on electrodes. Fabricating organic coatings for neural interface electrodes are largely laboratory‐based procedures with little information on industrial approaches. The fabrication of CHs differs based on the fabrication protocols of the two components (hydrogel and conductive component). Conductive components are introduced into the hydrogel matrix either by synthesis inside a prefabricated hydrogel or in parallel during the hydrogel cross‐linking reaction. Alternatively, the pre‐synthesized conductive component can be combined with the hydrogel precursors and integrated within the hydrogel matrix following its cross‐linking.^[^ ^254^ ^]^ For example, Gan et al., fabricated conductive, redox‐active, water‐soluble hydrogel via the self‐assembly of PEDOT on the polydopamine‐reduced and sulfonated graphene oxide (PSGO) template and incorporating them on polyacrylamide (PAM) adhesive hydrogel. The PSGO‐PEDOT‐PAM hydrogel showed the highest conductivity (108 S m^−1^), five times higher than the PSGO‐PAM hydrogel, with stable conductivity in the long run.^[^ ^255^ ^]^

Within laboratory‐based methods, the primary approaches to integrate CHs on electrode surfaces include chemical and electrochemical polymerization, spin‐coating, dip‐coating, and chemical vapor deposition. These techniques are used extensively in depositing CPs, CNTs, and graphene hydrogel coatings.^[^ ^92^, ^256^, ^257^ ^]^

Specifically, electrochemical polymerization holds particular favor in neural electrode coating due to the inherent stability of CPs in biological environments and the ease of application directly onto metals via electrodeposition. Electrodes coated either through spin coating, micropatterning, or electrochemical polymerization of PPy and PEDOT have shown great promise due to their excellent electrical properties, although the former exhibits less control over the thickness of the coated electrode, particularly in micron and sub‐micron size.^[^ ^125^, ^126^ ^]^

In situ fabrication is another approach in the manufacturing organic conductive coatings.^[^ ^258^, ^259^ ^]^ Strakosas et al. studied the electrode formation process within zebrafish and leech models by using naturally occurring metabolites to initiate enzymatic polymerization of a specific monomer called 2,5‐bis(2,3‐dihydrothieno[3,4‐b][1,4]dioxin‐5‐yl)thiophene acetic acid sodium salt which is based on trithiophene. This polymerization occurred within an injectable gel, creating CP gels that possess long‐range conductivity.^[^ ^259^ ^]^ In situ fabrication introduces novel possibilities for further exploration and development of organic conductive structures during manufacturing.

Despite advances in developing neural interfacing electrode coatings in laboratory settings, successful implementation of these technologies on an industrial scale for biomedical applications faces a series of challenges. Key considerations in the manufacturing process include the effect of coatings on electrode thickness, limited precision in controlling electrode dimensions during dip‐coating, the future requirement for coating the higher‐density array of electrodes, and the compatibility of materials with sterilization procedures used in the industry setting.^[^ ^260^ ^]^ In addition, coatings and manufacturing processes must not add significant biological risks to devices, which is considered in the next section.

### 4.3 Biocompatibility Considerations

Coatings have long been applied to metals used in industrial and consumer applications to improve their properties and enhance their functionality. In biomedical applications, metal coatings have also found wide acceptance in the cardiovascular and orthopaedic fields where metallic stents and artificial joints are coated to improve blood compatibility and osseointegration.^[^ ^261^, ^262^ ^]^ In neuromodulation applications, stringent requirements are placed on coating design due to the extreme demands of the biological environment combined with the need for maintenance of electrical stimulation over extended periods. Biological performance under these conditions requires robust cohesive and adhesive coatings that do not incite adverse host responses and must provide significant benefits over the metal electrode alone to be introduced into commercial devices.

**Figure 9:**
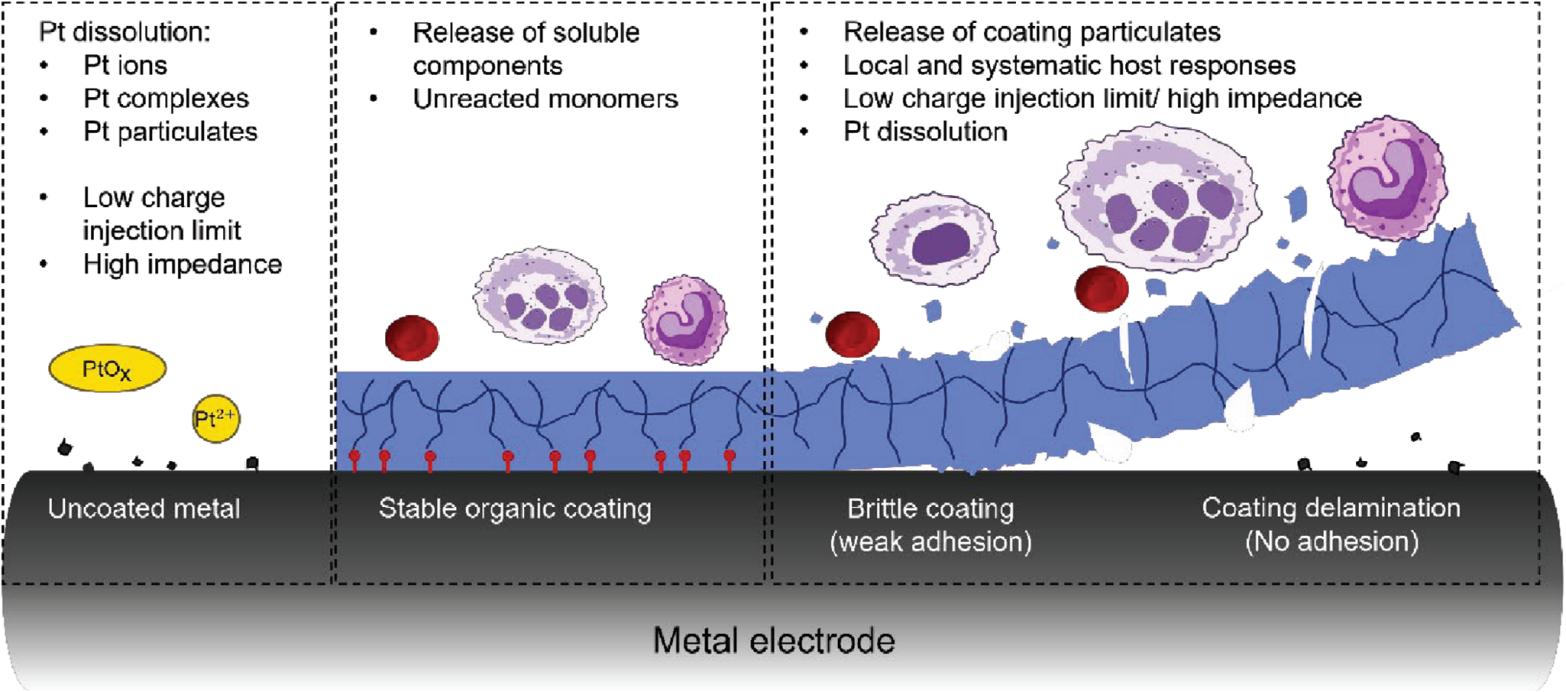
Schematic of the key challenges associated with coating bioelectrodes for neuromodulation applications and the potential impacts on host response.

Material responses to the host environment and the subsequent host responses depend on the nature and duration of contact and the type of tissue that is in contact with the device.^[^ ^263^ ^]^ Implantable neuromodulation electrodes tend to interface primarily with soft electrically active neural tissues such as in the brain, spinal cord and peripheral nerves. While devices may contact other tissues, like in the cochlear implant where there is some contact with bone, and spinal cord stimulators in which leads pass through soft tissues, electrode placement aims to be in close apposition to the target tissue. Thus, the primary interaction of electrode coatings is direct contact with nervous tissue. **Figure** **9** illustrates the material and host responses that may occur in coated bioelectrodes.

Optimal biological performance of an organic conductive coating relies on the inherent physical and chemical properties of the material as well as its interaction with the metal substrate. If the coating is stable with strong adhesion to the substrate, its physical, electrical and chemical properties will drive local tissue responses. These properties include physical geometry, topography, mechanical properties, and the chemical characteristics of the surface. While few papers have reported systematic preclinical evaluation of organic conductors, over the past two decades there has been a growing body of research supporting acceptable biological performance of CPs, hydrogels and composites.^[^ ^61^, ^88^, ^120^, ^264^ ^]^

Many studies of the cell and tissue interactions with polymer‐based conducting materials are on materials fabricated in research laboratories using a wide range of fabrication techniques. As noted previously, these laboratory‐fabricated materials are likely to release dopants which are not chemically bound to the polymer backbone; however, they have been shown to support neural cell growth in vitro and have acceptable tissue compatibility in vivo.^[^ ^78^, ^94^, ^95^, ^115^, ^116^ ^]^ Literature reports of commercially available CPs such as the proprietary PEDOT:PSS product, Clevios^TM^ suggests good biological performance however, there remain issues with coating stability as the base product is soluble and may require crosslinking to stabilize the coating.^[^ ^265^, ^266^, ^267^ ^]^ Any systemic responses resulting from soluble products released will be driven primarily by the chemical properties of the leachable components or degradation products. Appropriate preclinical biological testing plans need to identify and quantitate any leachable components and carefully analyze the risk of acute and chronic host responses.

Coating instability can result in leachable components as noted above, or the coating could undergo physical alteration and release polymeric particulates or even experience complete coating loss which may have broader local or systemic impacts. When coatings are intentionally degradable or constructed using textured, friable or brittle materials, material cohesion can be poor, compromising coating stability.^[^ ^120^, ^268^ ^]^ In this case, release of macroscopic or microscopic particulates due to coating instability can drive local and systemic host responses (Figure 9).

Exacerbation of tissue responses as a result of particles released from electrodes has been observed in post‐mortem clinical samples. Although these studies are limited in number, deterioration of cochlear electrode surfaces and release of Pt particulates from uncoated Pt electrodes during stimulation has been associated with higher levels of fibrotic tissue containing inflammatory and foreign body giant cells.^[^ ^3^, ^269^ ^]^ Animal studies of cortical implants have also linked deterioration of both the Pt electrodes and the insulating materials with increased fibrosis.^[^ ^270^ ^]^ In vivo studies of organic coatings such as CHs and CP‐CNTs composites have similarly suggested that coating disruption can release particles into the surrounding tissue, increasing the inflammatory response and fibrous tissue encapsulation,^[^ ^121^, ^271^ ^]^ underscoring the critical need for stable, adhesive coating strategies.

If adhesion to the underlying substrate is weak or becomes compromised, not only will the electrical properties deteriorate, but adverse tissue responses will likely occur. Such delamination of electrode coatings is a fundamental challenge that must be overcome so that the benefits of such coatings can be realized.^[^ ^24^ ^]^ As discussed, although there are many strategies for improving coating adhesion, their implementation into the clinic has not yet occurred.

While the risks of coating loss or delamination impacting device performance and host response must be carefully considered, these risks should be weighed against the potential risks of Pt electrode deterioration. In vitro studies suggest that both Pt nanoparticles and Pt ions are released from current devices and that they can impact biological performance in clinical and animal studies.^[^ ^272^ ^]^ If devices of the future look to improving resolution through increasing electrode numbers and decreasing electrode size the demands on Pt will more often exceed its capability to deliver the required charge densities and electrode deterioration will become a more significant challenge. This will open up further opportunities for coatings that have more robust charge transfer capabilities with higher safe levels of charge injection.

## 5 Conclusion

Conductive organic coatings have great potential to improve electrode function in neuromodulating applications. However, there remain significant challenges that need to be addressed before such organic coatings can be applied clinically. These relate to assuring coating stability and adhesion to underlying metal substrates, overcoming manufacturing challenges, and analyzing the biological risks and conducting appropriate preclinical evaluations.

Coating instability is one of the most critical barriers to the implementation of organic coatings in neuromodulating devices. Coatings that are unstable or that delaminate impact significantly on the electrical properties of the device. An essential design criterion for coatings is that they improve device functions over the substrate material, and in electrode applications, maintaining appropriate electrical function is a core requirement. Furthermore, when coatings deteriorate, soluble components and particulate release can exacerbate host responses which further impacts electrical properties and can result in the need for device removal.

It is therefore a fundamental requirement to understand adhesion mechanisms in biological environments over equivalent time periods to those for clinical use. Measuring coating stability in vitro and predicting in vivo performance remains challenging. Many of the standardized tests available are challenging to apply to clinical devices due to the small size and complex geometry of electrode arrays. Indirect testing methods encompassing accelerated mechanical disruption or extreme electrical conditions are promising but likely need to be tailored to each device type, adding significant complexity to standardization attempts.

Most manufacturing processes developed for organic coatings arise from research laboratories and are difficult to translate into commercial settings. Current commercial electrode arrays are typically fabricated using manual processes by highly trained personnel. Major challenges in translating laboratory approaches into products include limited precision in controlling electrode dimensions, compatibility of coating materials with sterilization procedures and the potential for coating processes to impact on other components of the array. To receive regulatory approval, it is critical for any new manufacturing process to assure that it will not affect device electrical or biological performance.

Demonstrating appropriate material and host responses is a risk‐based process requiring development of a biological evaluation plan. This plan must consider the risks and how they will be mitigated through a combination of prior knowledge, laboratory tests and preclinical animal studies. Each type of organic coating will have different chemistries and physical/mechanical properties meaning that the biological performance tests must be tailored to the specific material and application. Apart from coating instability, which is discussed above, there are several more generic aspects of material and host responses to organic coatings that remain unknown. These include the degree of leachable components such as mobile dopants and monomers likely to be released, and the host responses to these, and the long‐term electrical performance of coatings in vivo.

As the neuromodulation field experiences a paradigm shift due to new requirements driven by miniaturized and minimally invasive device designs, a preferred path for advancing technologies lies in creating innovations that can easily integrate with established biomedical materials. This will likely accelerate progress in understanding adhesion mechanisms, improving coating stability and increasing knowledge on the long‐term in vivo performance of organic bioelectronic materials.

## Conflict of Interest

The authors declare no conflict of interest.
